# EBF1 binds to EBNA2 and promotes the assembly of EBNA2 chromatin complexes in B cells

**DOI:** 10.1371/journal.ppat.1006664

**Published:** 2017-10-02

**Authors:** Laura V. Glaser, Simone Rieger, Sybille Thumann, Sophie Beer, Cornelia Kuklik-Roos, Dietmar E. Martin, Kerstin C. Maier, Marie L. Harth-Hertle, Björn Grüning, Rolf Backofen, Stefan Krebs, Helmut Blum, Ralf Zimmer, Florian Erhard, Bettina Kempkes

**Affiliations:** 1 Department of Gene Vectors, Helmholtz Center Munich, Munich, Germany; 2 Gene Center, Ludwig-Maximilians-University, Munich, Germany; 3 Bioinformatics, Institute for Informatics, Albert-Ludwigs-University, Freiburg, Germany; 4 Teaching and Research Unit Bioinformatics, Institute of Informatics, Ludwig-Maximilians-University, Munich, Germany; Baylor College of Medicine, UNITED STATES

## Abstract

Epstein-Barr virus (EBV) infection converts resting human B cells into permanently proliferating lymphoblastoid cell lines (LCLs). The Epstein-Barr virus nuclear antigen 2 (EBNA2) plays a key role in this process. It preferentially binds to B cell enhancers and establishes a specific viral and cellular gene expression program in LCLs. The cellular DNA binding factor CBF1/CSL serves as a sequence specific chromatin anchor for EBNA2. The ubiquitous expression of this highly conserved protein raises the question whether additional cellular factors might determine EBNA2 chromatin binding selectively in B cells. Here we used CBF1 deficient B cells to identify cellular genes up or downregulated by EBNA2 as well as CBF1 independent EBNA2 chromatin binding sites. Apparently, CBF1 independent EBNA2 target genes and chromatin binding sites can be identified but are less frequent than CBF1 dependent EBNA2 functions. CBF1 independent EBNA2 binding sites are highly enriched for EBF1 binding motifs. We show that EBNA2 binds to EBF1 via its N-terminal domain. CBF1 proficient and deficient B cells require EBF1 to bind to CBF1 independent binding sites. Our results identify EBF1 as a co-factor of EBNA2 which conveys B cell specificity to EBNA2.

## Author summary

Epstein-Barr virus (EBV) infection is closely linked to cancer development. At particular risk are immunocompromised individuals like post-transplant patients which can develop B cell lymphomas. In healthy individuals EBV preferentially infects B cells and establishes a latent infection without causing apparent clinical symptoms in most cases. Upon infection, Epstein-Barr virus nuclear antigen 2 (EBNA2) initiates a B cell specific gene expression program that causes activation and proliferation of the infected cells. EBNA2 is a transcription factor well known to use a cellular protein, CBF1/CSL, as a DNA adaptor. CBF1/CSL is a sequence specific DNA binding protein robustly expressed in all tissues. Here we show that EBNA2 can form complexes with early B cell factor 1 (EBF1), a B cell specific DNA binding transcription factor, and EBF1 stabilizes EBNA2 chromatin binding. This EBNA2/EBF1 complex might serve as a novel target to develop future small molecule strategies that act as antivirals in latent B cell infection.

## Introduction

CBF1/CSL (C promoter binding factor, Suppressor of Hairless, and lag1 also called RBPJ or RBPJκ) is a cellular DNA binding protein, ubiquitously expressed in all mammalian tissues. CBF1 serves as a DNA adaptor molecule that recruits either repressors or activators to transcriptional control elements like enhancers and transcription start sites of genes and is described as the major downstream effector of the cellular Notch signal transduction pathway [1]. Notch signaling controls the development and differentiation of diverse organs and tissues. Despite the ubiquitous expression of its chromatin anchor CBF1, target gene control by Notch is context dependent and requires tissue and lineage specific cooperating transcription factors [2]. In B cells, latently infected with Epstein-Barr virus (EBV), CBF1 anchors the viral transactivator protein EBV nuclear antigen 2 (EBNA2) to chromatin and thereby initiates a cascade of signaling events that coordinate B cell activation and proliferation of infected cells [3–6]. Thus, EBNA2 is considered to mimic Notch signaling [7]. In contrast to the universal expression and pleiotropic activities of Notch, the expression and the biological activity of EBNA2 is strictly confined to EBV infected B cells, characterized by a transcription program that phenocopies antigen activated B cell blasts [8, 9].

CBF1 and EBNA2 frequently co-occupy cellular enhancer and super-enhancer regions reinforcing the concept that CBF1 is the major adaptor for EBNA2 to chromatin [10]. In addition, EBNA2 bound regions are co-occupied with multiple additional transcription factors including IRF4, BATF, NFκB, Runx, and ETS family members as well as the B cell lineage defining and pioneer factors PU.1/SPI1 and EBF1 [10, 11]. While the adaptor function of CBF1 is well defined, a potential functional contribution of these co-occurring factors to EBNA2 function has not been studied thoroughly. These proteins are active transcription factors which carry transactivation domains and can actively promote or impair transcription of target genes. PU.1/SPI1 promotes B cell development and is expressed throughout B cell differentiation, but also controls T cell, myeloid and dendritic cell differentiation [12]. PU.1/SPI1 DNA binding sites are critical for LMP1 promoter luciferase activation [3, 13–15]. However, its contribution to LMP1 expression in the context of the entire viral genome is surprisingly weak [16]. Most recently it has been shown that EBNA2 enhances the binding of CBF1 and EBF1 to chromatin and EBF1 is critical for expression of the EBNA2 viral target gene LMP1 [16, 17]. By sequential chromatin immunoprecipitation, EBF1 and EBNA2 have been shown to bind to the same chromatin fragment in the same cell [17]. Importantly, within the hematopoietic compartment EBF1 is exclusively expressed in B cells and their lymphocytic precursors. The other EBF gene family members EBF2, 3, and 4 are expressed at very low or undetectable levels in B cells. EBF1 initiates B cell lineage commitment, development and differentiation as a pioneer factor that promotes chromatin accessibility and DNA demethylation in lymphocyte precursors [18, 19].

Strong EBNA2 binding correlates with extended regions of extraordinarily high histone 3 lysine 27 acetylation (H3K27ac) and H3K4 mono-methylation (H3K4me1) marks which are characteristic features of activated super enhancers [11]. In addition, EBNA2 modulates the formation of chromatin loops to connect enhancers and promoters of its target genes [20]. In theory, EBNA2 co-occurring factors, like PU.1/SPI1 and EBF1 could function as pioneer factors for EBNA2 by modulating the chromatin state and thereby promoting access of EBNA2 to chromatin, indirectly. Alternatively, EBNA2 co-occurring factors might serve as alternate adaptors that promote DNA binding of EBNA2.

CBF1 is ubiquitously expressed in all mammalian cells including primary human B cells and EBV infected and non-infected human B cell lines. For this study, we used a CBF1 deficient human B cell line, which had been generated by homologous recombination in the somatic B cell line DG75, to screen for CBF1 independent functions of EBNA2. The parental DG75 B cell line is an EBV negative Burkitt's lymphoma cell line. While the knock-down of EBF1 and CBF1 in EBV immortalized B cells severely impairs cellular viability [17], DG75 cells tolerate inactivation of the CBF1 gene without loss of viability [21, 22]. We compared EBNA2 induced cellular genes in CBF1 proficient and deficient DG75 cells and found the majority of EBNA2 target genes to be CBF1 dependent. A minor fraction of EBNA2 target genes is regulated CBF1 independently. By chromatin immunoprecipitation and genome wide sequencing of EBNA2 bound DNA fragments (ChIP-Seq), we identified a subpopulation of CBF1 independent EBNA2 binding sites that was significantly enriched for EBF1 binding motifs. We show that CBF1 independent EBNA2 binding to chromatin is dependent on EBF1 protein expression. Importantly, we demonstrate that EBNA2 and EBF1 can form protein complexes in CBF1 positive and negative cells, indicating that EBF1 serves as B cell specific DNA anchor for EBNA2.

## Results

### Genome wide expression profiling identifies cellular transcripts regulated by EBNA2 in CBF1 deficient B cells

**Fig 1:**
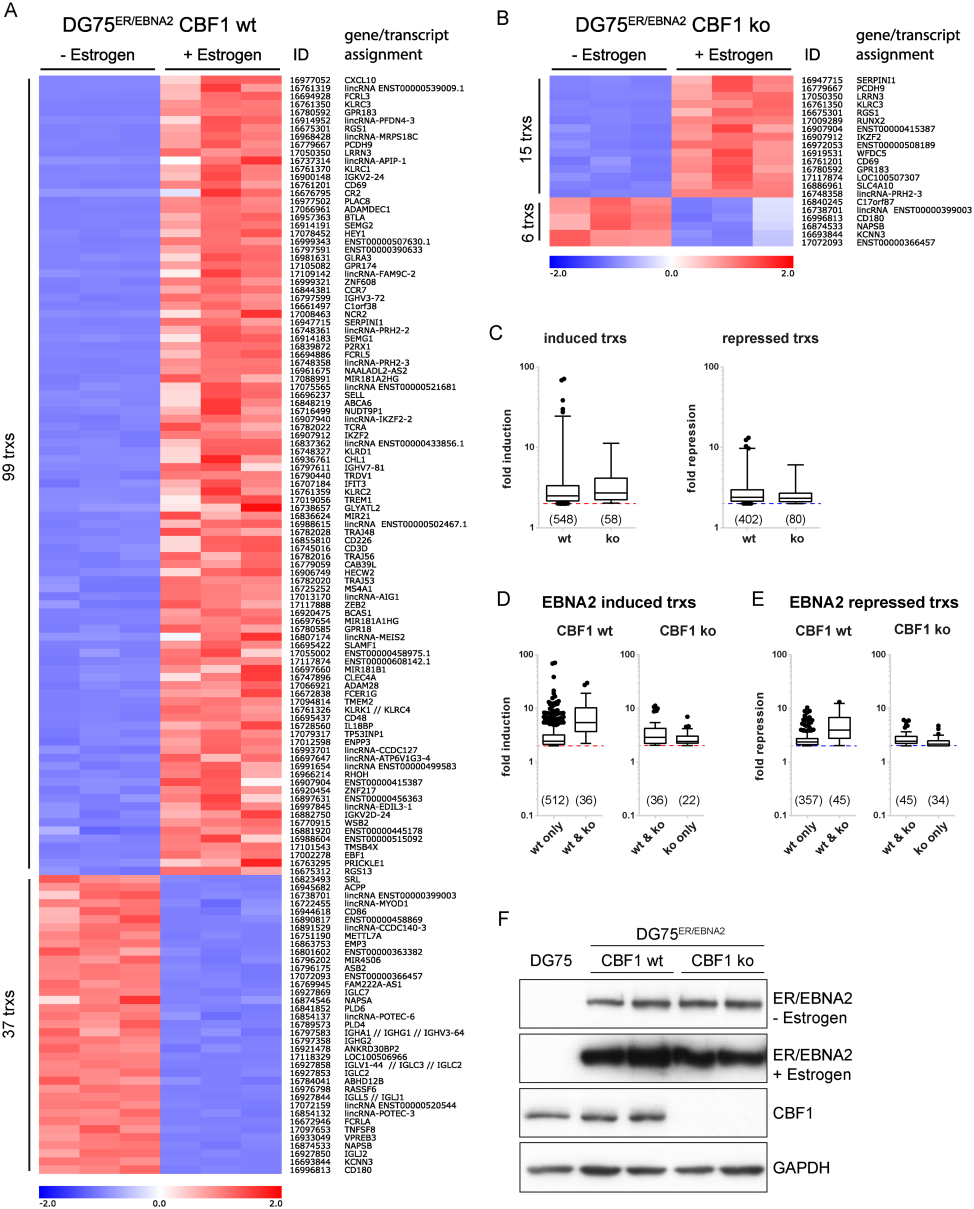
Comparative transcript profiling of EBNA2 target gene expression in CBF1 proficient and deficient DG75 cells. DG75 cells expressing ER/EBNA2 were cultivated in estrogen supplemented medium for 24 h or were left untreated. Total cellular RNA was isolated and submitted to gene expression analysis using the Human Gene 2.0 ST array. All probe sets represent single transcripts (trxs). For each condition, 3 biological replicates were examined. Each vertical column represents the results obtained after hybridizing a single microarray. Horizontal rows represent data obtained for a particular probe set across all cell lines and conditions adjusted to a scale ranging from -2.0 to + 2.0. The relative high, medium and low expression values are represented by red, white and blue color, respectively. Vertical columns are ranked according to fold changes from highest induction levels on top to highest repression levels at the bottom. (A) Expression levels of 136 transcripts which change expression levels at least 4-fold (p ≤ 0.001) in response to EBNA2 in CBF1 proficient DG75 (DG75^ER/EBNA2^ CBF1 wt) cells are displayed. The transcript cluster ID and the assigned genes/transcripts, including non-coding RNAs, are annotated. (B) 21 transcripts regulated at least 4-fold (p ≤ 0.001) in CBF1 deficient DG75 (DG75^ER/EBNA2^ CBF1 ko). (C) Boxplots depicting the fold change distribution of EBNA2 induced and repressed transcripts for the subset of target genes changed at least 2-fold (p ≤ 0.05) in CBF1 wt and ko cells, respectively. EBNA2 induced (D) and repressed (E) transcripts are shown to illustrate the dynamic range of each system. Boxplot whiskers extend to 1.5x interquartile range. Dotted lines mark the 2-fold change chosen as cut-off. (F) Expression levels of EBNA2 (prior to and after estrogen treatment) and CBF1 proteins were monitored by Western blot analysis. Equal amounts of total protein lysates were applied and GAPDH served as an internal loading control. One representative experiment (n = 3) is shown.

In order to rigorously test if EBNA2 can exert any functions in the absence of its DNA adaptor CBF1, a microarray based genome wide screen for EBNA2 target genes in DG75 B cells that are either proficient (wt) or deficient (ko) for CBF1 was performed (Fig 1A, 1B and 1F and S1 Table). Both cell lines constitutively express an estrogen receptor (ER) hormone binding domain EBNA2 fusion protein (ER/EBNA2). ER/EBNA2 is retained in the cytoplasm of the cell but is rapidly activated and translocated to the nucleus in response to estrogen [21, 22]. For expression profiling, DG75^ER/EBNA2^ CBF1 wt and CBF1 ko cells were cultured in estrogen supplemented media for 24 h, total cellular RNAs were harvested and processed for the hybridization of gene arrays that detect 30645 coding transcripts, 11086 lincRNAs (long intergenic non-coding RNA transcripts) and 148 miRNAs (micro RNAs). Cell cultures of the parental DG75 CBF1 wt and CBF1 ko cell lines, which do not express ER/EBNA2, were treated with estrogen and processed for the microarray analysis as specificity controls. Neither in DG75 CBF1 wt nor in DG75 CBF1 ko cells statistically significant changes (p ≤ 0.05) of cellular transcript abundance in response to estrogen treatment were observed, proving that target gene activation is strictly dependent on ER/EBNA2 (S1A Fig). In addition, estrogen responsive target genes described in the literature did not change expression levels proving that the estrogen receptor response is not functional in DG75 B cells (S1B Fig) [23–27]. It is important to note that EBNA2 not only activates a set of direct target genes but thereby initiates a cascade of secondary events, which are included in our target gene lists and in total reflect EBNA2 functions.

Based on expression level changes of 950 transcripts (≥ 2-fold, p≤ 0.05) which are regulated in DG75^ER/EBNA2^ CBF1 wt and expression levels of the same transcripts in DG75^ER/EBNA2^ CBF1 ko cells 12 clusters of transcripts were defined and illustrate the complex patterns that arise. Transcripts in cluster I, II, IV and VI are upregulated in CBF1 wt cells. Cluster VII transcripts are upregulated in both, wt and ko cells. Cluster III transcripts are down-regulated in CBF1 wt only, while cluster VIII transcripts are down-regulated in both, wt and ko cell (S2 Fig and S2 Table). Multiple previously characterized EBNA2 target genes were significantly upregulated in DG75^ER/EBNA2^ CBF1 wt cells (S1C Fig). In total, 99 cellular transcripts were up- and 37 cellular transcripts were downregulated ≥ 4-fold (p ≤ 0.001) (Fig 1A). Importantly, 15 transcripts were upregulated and 6 transcripts were downregulated in CBF1 deficient DG75^ER/EBNA2^ ≥ 4-fold (Fig 1B). Although the number of differentially expressed EBNA2 target genes was markedly higher in CBF1 proficient cells, the mean changes of the response were similar in CBF1 proficient and deficient cells as illustrated for genes regulated ≥ 2-fold (p ≤ 0.05) (Fig 1C).

The majority of EBNA2 target genes identified in CBF1 deficient cells were also regulated in CBF1 proficient cells, while a small group of targets is regulated by EBNA2 in CBF1 deficient cells, only (ko only, Fig 1D and 1E). On average, the transcripts which are regulated in CBF1 proficient and deficient cells (wt & ko) showed a stronger response than those regulated in proficient cells, only (wt only). In order to verify the microarray results, a panel of 12 CBF1 dependent (S2 Fig cluster 2) and independent targets (S2 Fig cluster 7) was selected for re-testing. RT-qPCR experiments confirmed that most CBF1 independent targets also responded to EBNA2 in CBF1 proficient cells. As already seen in the microarray experiment, the degree to which individual targets responded in CBF1 proficient cells varied considerably but was faithfully reproduced by RT-qPCR (S4 Fig). Interestingly, the CBF1 dependent target genes included a substantial number of miRNAs that are up- or downregulated by EBNA2 (S5 Fig).

### CBF1 independent EBNA2 repressed target genes are enriched for genes involved in B cell signaling

To functionally characterize EBNA2 target genes, biological processes associated with individual subsets of genes were analyzed. The online tool GOrilla was used to test whether differentially expressed genes in comparison to all other genes on the array were enriched in any of the GO terms in the “Biological Process” category. The subsets considered here consisted of genes that were on average induced or repressed in the CBF1 proficient and deficient cell lines, or genes where induction or repression was dependent or independent of CBF1. Only genes significantly (q < 0.01) regulated in at least one of the two cell lines were considered. Thresholds on fold changes were chosen by the online tool GOrilla in a data dependent manner to identify subsets enriched in GO terms in the “Biological Process” category (S6 Fig).

Neither genes repressed in CBF1 proficient cells only (repressed/CBF1 dependent) nor induced in CBF1 deficient cells (induced/CBF1 independent) were significantly (q ≤ 10^−4^) enriched for any biological process. Genes induced in CBF1 proficient cells only (induced/CBF1 dependent) were strongly and most significantly enriched for immunoglobulin receptor binding and moderately enriched for biological processes involving several enzymatic activities (Table 1).

**Table 1:** Gene ontology enrichment analysis of CBF1 dependent EBNA2 induced target genes.

Term ID*	Term	Genes in term	Target genes in term**	Enrichment Score***	q-value
GO:0034987	immunoglobulin receptor binding	19	10	58,08	5,59E-12
GO:0004252	serine-type endopeptidase activity	30	8	29,43	1,13E-06
GO:0008236	serine-type peptidase activity	33	8	26,75	2,16E-06
GO:0017171	serine hydrolase activity	33	8	26,75	1,80E-06
GO:0003823	antigen binding	49	11	24,77	5,03E-09
GO:0070011	peptidase activity, acting on L-amino acid peptides	137	8	15,39	1,14E-04
GO:0008233	peptidase activity	142	8	14,85	1,35E-04
GO:0004175	endopeptidase activity	84	12	12,79	1,25E-06
GO:0004872	receptor activity	226	33	2,61	6,65E-04
GO:0060089	molecular transducer activity	226	33	2,61	5,98E-04

*The top 10 GO terms in the “Biological Process” category are depicted. Note that a given gene can be annotated to multiple terms.

**number of genes in the top of the EBNA2 target gene list (chosen by GOrilla)

***Enrichment of a given GO term among differentially regulated genes with respect to the total number of genes assayed and annotated to them, calculated by GOrilla, see Material and methods

Target genes repressed by EBNA2 in the absence of CBF1 (repressed/ CBF1 independent) showed a remarkable profile (Table 2). They map to several GO terms that cover diverse immune responses. Since the study had been performed in B cells, the enrichment for genes involved in immune responses and B cell receptor biology could have been expected. However, our study indicates that EBNA2 also represses immune response genes and this feature of EBNA2 is CBF1 independent.

**Table 2:** Gene ontology enrichment analysis for CBF1 independent EBNA2 repressed target genes.

Term ID*	Term	Genes in term	Target genes in term**	Enrichment Score***	q-value
GO:0002768	immune response-reg. cell surface receptor signaling pathway	46	27	3,52	1,09E-06
GO:0002757	immune response-activating signal transduction	52	30	3,46	3,87E-07
GO:0002764	immune response-reg. signaling pathway	54	31	3,44	3,08E-07
GO:0050778	positive regulation of immune response	68	36	3,18	2,96E-07
GO:0002429	immune response-act. cell surface receptor signaling pathway	44	31	2,87	1,29E-06
GO:0050776	regulation of immune response	84	39	2,79	1,25E-06
GO:0002253	activation of immune response	54	36	2,72	8,67E-07
GO:0002376	immune system process	149	56	2,26	1,16E-06
GO:0007166	cell surface receptor signaling pathway	152	73	1,9	1,90E-06
GO:0007165	signal transduction	311	128	1,59	6,01E-07

*The top 10 GO terms in the “Biological Process” category are depicted. Note that a given gene can be annotated to multiple terms.

**number of genes in the top of the EBNA2 target gene list (chosen by GOrilla)

***Enrichment of a given GO term among differentially regulated genes with respect to the total number of genes assayed and annotated to them, calculated by GOrilla, see Material and methods

### EBNA2 is recruited to chromatin in CBF1 deficient B cells

**Fig 2:**
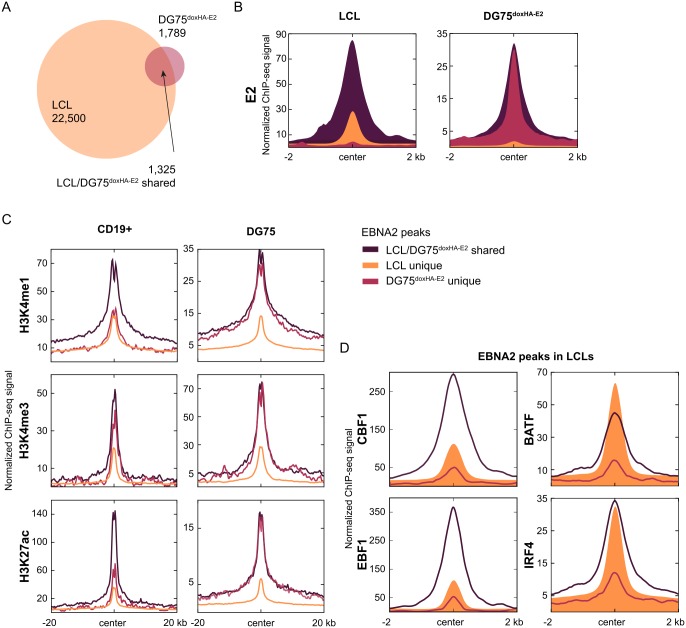
Cell line specific chromatin signatures predispose specific sites for EBNA2 binding. (A) Intersection of EBNA2 binding sites identified in LCLs and DG75^doxHA-E2^ CBF1 wt cells. (B) Anchor plots showing EBNA2 ChIP-seq signal intensities for LCLs and for DG75^doxHA-E2^ CBF1 wt at sites identified in both cell lines (LCL/DG75^doxHA-E2^ shared) or unique to either cell line (LCL unique or DG75^doxHA-E2^ unique). (C) ChIP-seq signals associated with active chromatin and enhancer state (H3K4me1, H3K4me3, and H3K27ac) at EBNA2 binding sites in CD19+ B cells and DG75 cell line. Using ChIP-seq data provided by public resources [28, 29], the mean normalized signal for each histone modification and peak subset was calculated for the region flanking all EBNA2 peak centers for 20 kb in each direction, applying the same workflow for CD19+ B cells and DG75 data sets. Please note that absolute values of signal intensities for the same histone modification should not be compared between the two cell lines since the experiments were conducted by different laboratories using different antibodies. (D) ChIP-seq signals for CBF1, EBF1, BATF and IRF4 at LCL/DG75^doxHA-E2^ shared or LCL unique or DG75^doxHA-E2^ EBNA2 binding peaks.

In summary, our differential expression analysis of EBNA2 target genes shows that EBNA2 can regulate a small fraction of its target genes without using CBF1 as a DNA anchor. In order to uncover alternative strategies of EBNA2 to bind to chromatin, we performed chromatin immunoprecipitation (ChIP) studies to identify genomic loci that are bound by EBNA2 in CBF1 negative cells. In ER/EBNA2 expressing cells, EBNA2 shuttles from the cytoplasm to the nucleus in response to estrogen. In order to avoid a potential impact of cytoplasmic ER/EBNA2 contamination on our biochemical studies, we switched to a doxycycline inducible HA-EBNA2 expression system (doxHA-E2) in DG75 (S7A Fig). In the absence of doxycycline, EBNA2 is not expressed and cannot interfere with the immunoprecipitation procedure in DG75^doxHA-E2^/CBF1 wt and DG75^doxHA-E2^/CBF1 ko cells. Up to 90% of the cells express EBNA2 when treated with doxycycline (S7B Fig). EBNA2 protein signal detected by immunostaining was 5- to10-fold stronger than EBNA2 in LCLs (S7C Fig). In comparison to LCLs, some of the EBNA2 co-occurring transcription factors like BATF and IRF4 were expressed at very low levels while EBF1 and PU.1/SPI1 were robustly expressed (S7C Fig). ChIP followed by high throughput sequencing (ChIP-seq) was performed to determine EBNA2 genome occupancy. 1,789 EBNA2 binding sites were identified in CBF1 proficient DG75^dox-HA2^ (Fig 2A), while 22,500 EBNA2 peaks were identified in LCLs, which had been performed in parallel. 1,325 (74%) of the EBNA2 peaks in DG75^doxHA-E2^ cells were also present in LCLs (shared peaks), while 464 binding sites occurred exclusively in DG75^doxHA-E2^ cells. EBNA2 signal intensity was most prominent at LCL/DG75^doxHA-E2^ shared EBNA2 binding sites (Fig 2B and S8 Fig).

In LCLs, EBNA2 is preferentially recruited to enhancer elements which pre-exist in peripheral CD19 positive B cells before they are infected by EBV to generate LCLs [10]. Chromatin marks characteristic for activated enhancer elements are H3K27ac in combination with H3K4me1 signals that are stronger than H3K4me3. We speculated that DG75 specific chromatin signatures in the absence of EBV infection might influence EBNA2 binding. We thus compared H3K4me1, H3K4me3, and H3K27ac signal intensities at EBNA2 binding sites i) shared by LCLs and DG75^doxHA-E2^, ii) unique for LCLs and iii) unique for DG75 in naïve CD19 positive B cells with those in non-transfected DG75.

EBNA2 binding sites, shared by LCLs and DG75^doxHA-E2^, stand out as the subset with the most prominent enrichment for all three investigated histone modifications associated with the chromatin state of active enhancers (Fig 2C). In contrast, DG75^doxHA-E2^ unique EBNA2 binding sites were highly enriched for active chromatin marks in the DG75 precursor only, while LCL unique EBNA2 peaks showed significantly lower signal intensities in DG75. These data indicate that a set of enhancers, which are pre-activated in DG75 cells but not in the CD19 positive LCL precursors, might allow the formation of "DG75 unique" EBNA2 binding sites. DG75 lack pre-formed enhancer signatures at "LCL unique" binding sites. ChIP signals for CBF1 and EBF1 are most highly enriched at EBNA2 binding peaks shared by LCLs and DG75^doxHA-E2^, while enrichment at LCL unique peaks was attenuated. In contrast, BATF and IRF4 were strongly enriched at shared LCLs and DG75^doxHA-E2^as well as LCL unique peaks. Thus, low abundance of IRF4 and BATF proteins or other co-occurring transcription factors in DG75^doxHA-E2^ might limit EBNA2 occupancy in DG75 at these LCL unique sites.

**Fig 3:**
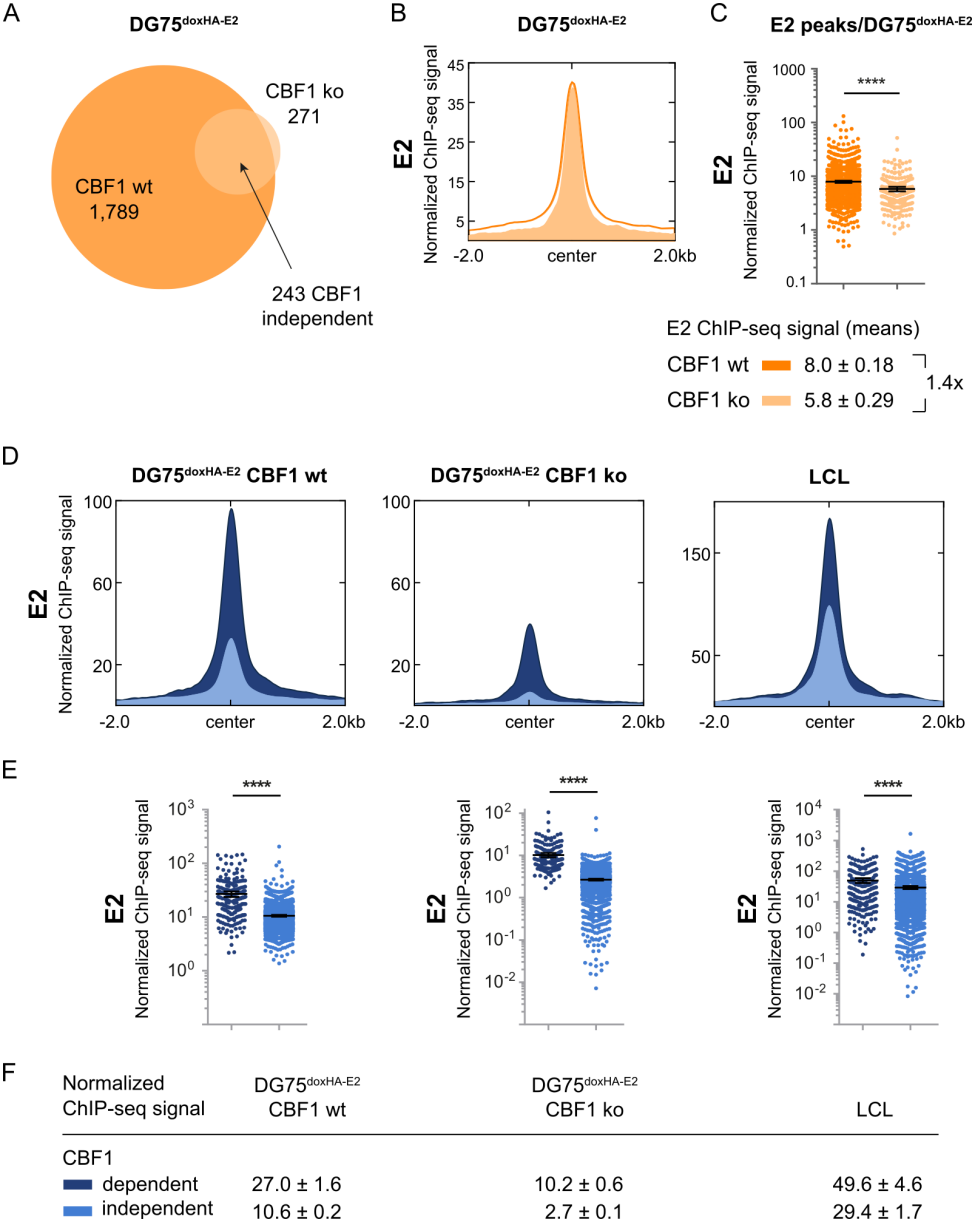
EBNA2 can access more than 15% of its chromatin binding sites in CBF1 deficient DG75 B cells. (A) Intersection of EBNA2 binding sites identified in CBF1 proficient or deficient cells 24 h post doxycycline induction. 1,546 peaks that were identified in CBF1 proficient but not in CBF1 deficient cells were defined as "CBF1 dependent" EBNA2 peaks. 243 EBNA2 peaks identified in CBF1 deficient and proficient DG75 cells were defined as "CBF1 independent". (B-E) Comparison of EBNA2 ChIP-seq signal distributions at CBF1 independent or dependent peaks. (B) Anchor and (C) scatter plots (mean + 95% CI) depicting ChIP-seq signal distributions at EBNA2 peak subsets. Regions flanking the peak center for 2 kb in each direction were analyzed (Data underlying panel B). Absolute means and SEMs are indicated below. (D) Anchor and (E) scatter plots (mean + 95% CI) as shown in B and C but depicting EBNA2 ChIP-seq signal intensities for the two different subsets of EBNA2 peaks as defined in A. Statistical significance for differences of all means were assessed applying unpaired two-tailed t-test for log values with Welch’s correction (**** p < 0.0001); absolute means and SEMs are indicated below. (F) List of EBNA2 mean ChIP-seq signal intensities at CBF1 independent and dependent peaks.

The comparison of ChIP-Seq data between DG75^doxHA-E2^ CBF1 wt and ko cells identified 1,789 EBNA2 binding sites in CBF1 proficient and 271 in CBF1 deficient DG75. 243 (81%) were found in both cell lines and thus constitute CBF1 independent EBNA2 peaks (Fig 3A). The majority of EBNA2 binding sites found in CBF1 proficient (74%) and deficient (83%) DG75 were shared with LCLs (S8 Fig). A small group of 28 binding sites were only identified in CBF1 deficient cells and were not analyzed further. 1,546 EBNA2 sites were not detected in CBF1 deficient cells and thus defined as "CBF1 dependent". The mean EBNA2 signal intensity at EBNA2 binding sites was elevated 1.4-fold in wt compared to ko cells (Fig 3B and 3C). Remarkably, EBNA2 binding to CBF1 independent peaks was significantly enriched compared to CBF1 dependent peaks in CBF1 wt cells (2.5-fold, Fig 3D and 3E). The quantitative re-analysis of the subclasses of EBNA2 peaks in LCLs confirmed that CBF1 independent peaks are characterized by stronger EBNA2 enrichment (Fig 3D, 3E and 3F, right panel). Since CBF1 independent EBNA2 binding obviously contributes to EBNA2 occupancy in LCLs, we conclude that our CBF1 deficient B cell line is a valid model system to study mechanisms which drive EBNA2 chromatin interactions.

**Fig 4:**
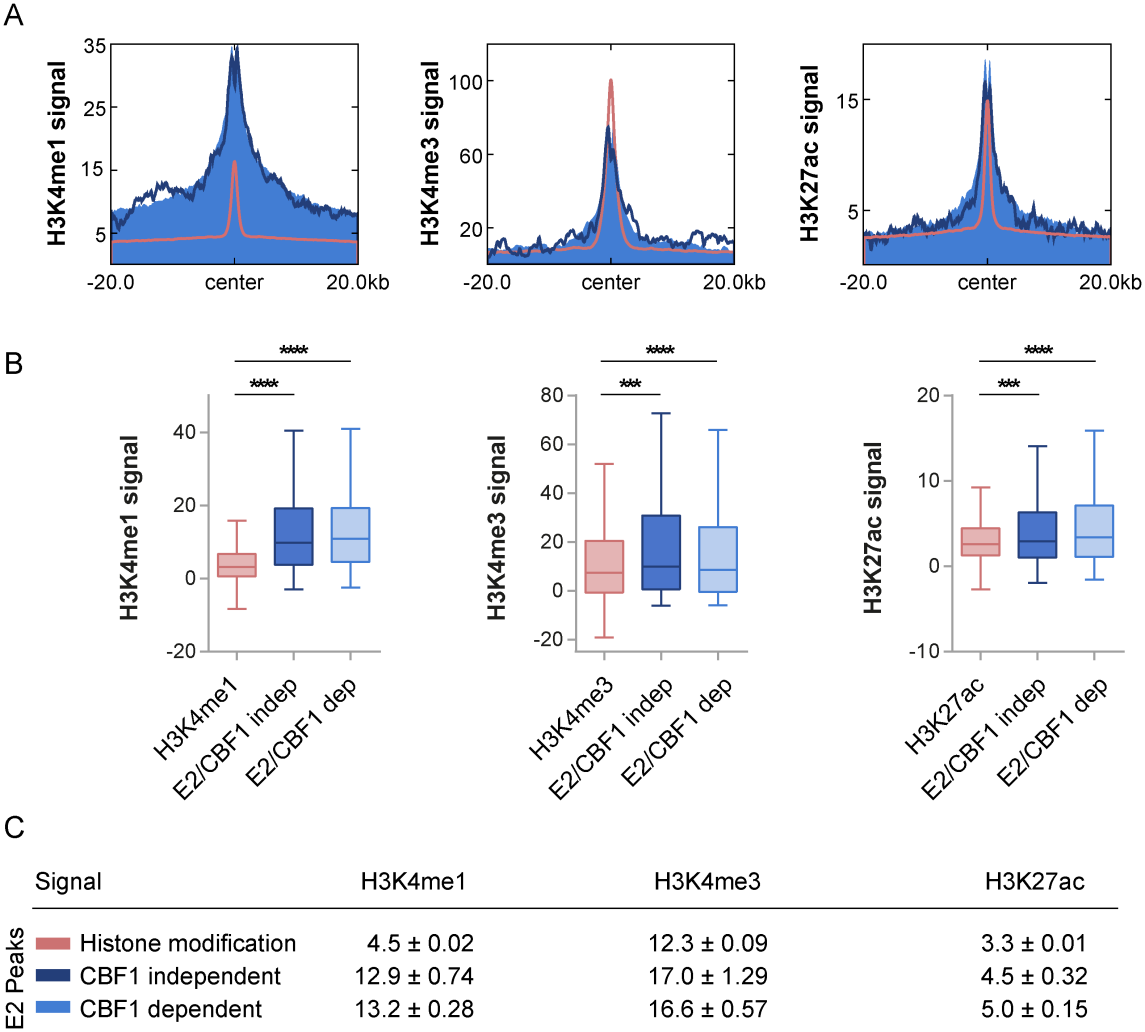
CBF1 independent and dependent EBNA2 binding sites are significantly enriched for activated chromatin marks in DG75 cells prior to EBNA2 binding. Based on published data sets on histone modification in DG75, the two EBNA2 peak subsets (CBF1 independent dark blue; CBF1 dependent light blue) were separately analyzed for histone activation marks typically found at enhancer regions. These data were compared to signal intensities of all peaks for the respective chromatin modification (red). (A) Anchor plots depict H3K4me1, H3K4me3, and H3K27ac at the respective peak centers and 20 kb flanking regions. (B) Data underlying panel (A) were used to generate boxplots showing the signal distributions encompassing the entire 40 kb genomic region. The significance of differences of means was assessed by unpaired two-tailed t-tests with Welch’s correction (**** p < 0.0001, *** p < 0.001). The differences of means for CBF1 independent compared to CBF1 dependent EBNA2 peaks for H3K4me1 (-0.3004 ± 0.7957; p = 0.706), H3K4me3 (0.4323 ± 1.411; p = 0.7595), and H3K27ac (-0.5184 ± 0.3501: p = 0.1396) were not statistically significant. Box plot whiskers extend to 1.5x interquartile range. (C) Table summarizing means and SEMs of histone modifications analyzed in (A) and (B).

To better characterize CBF1 dependent and independent EBNA2 binding sites prior to EBNA2 binding we could use H3K4me1, H3K4me3, and H3K27ac ChIP-Seq data published for DG75 [29]. Signal intensities of H3K4me1, H3K4me3, and H3K27ac were reanalyzed separately plotted for the CBF1 dependent and independent peak subpopulations and compared to the mean signal peak intensities of the respective chromatin modification in DG75 (Fig 4). All three activation marks showed almost the same high enrichment profiles for both subpopulations indicating that chromatin signatures are most probably not the trigger either for CBF1 dependent or independent binding.

### CBF1 independent EBNA2 peaks are significantly enriched for EBF1 binding motifs and EBF1 signal intensities in LCLs

**Fig 5:**
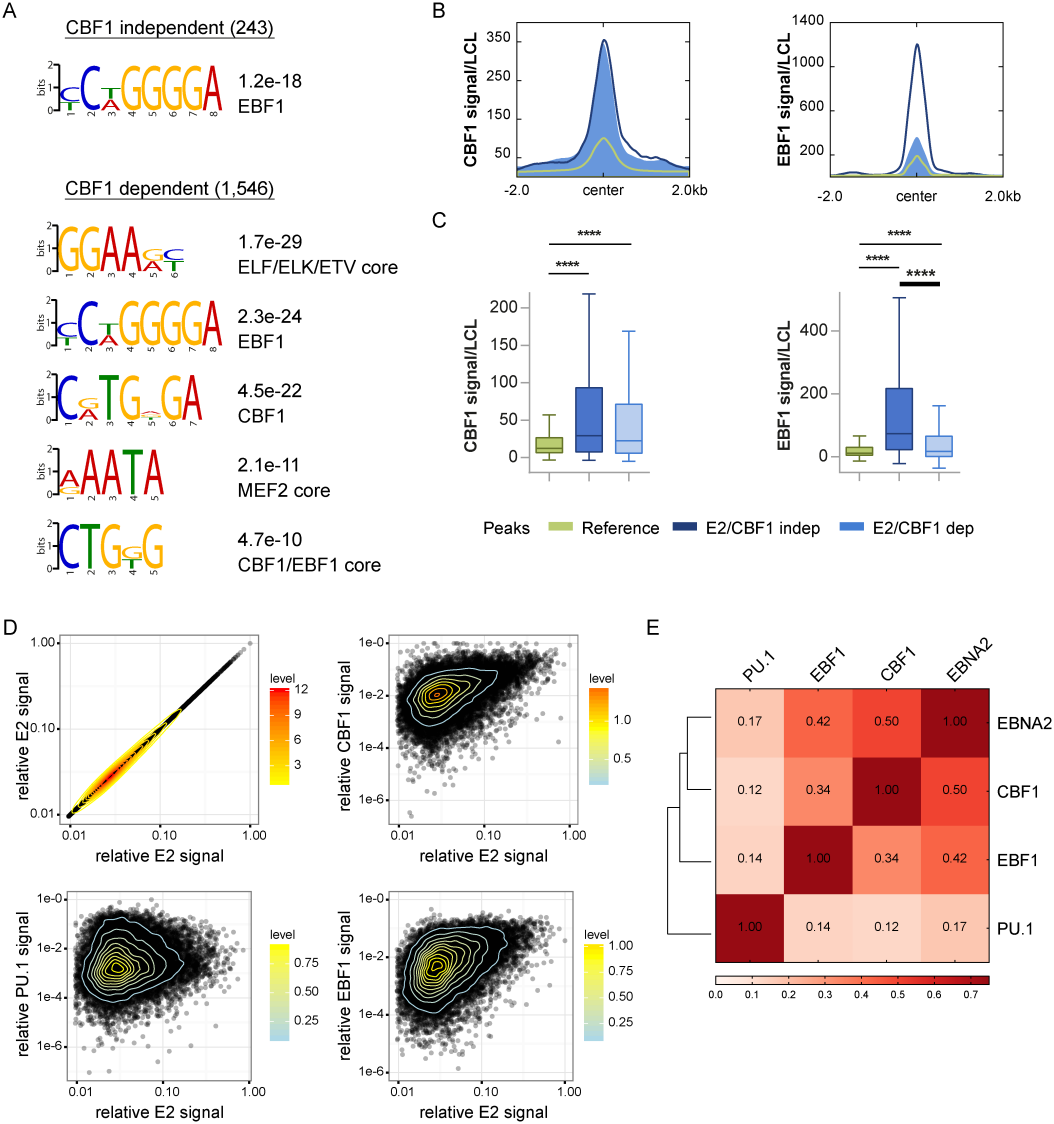
The EBF1 binding motif is highly enriched at CBF1 independent binding sites and the EBF1 signal correlates with EBNA2 binding signal distributions. (A) *De novo* identified DNA sequence motifs and the respective E-values at CBF1 independent and dependent EBNA2 binding sites as discovered by MEME-ChIP [30]. The analysis was performed for different sized data sets. TFs predicted to recognize the respective motifs, as assigned by TOMTOM (using the hocomoco v10 data base), are listed. If multiple TFs with comparable significances were assigned to one motif, the motif was designated as “core motif” for this subset. (B) CBF1 independent (dark blue) and dependent (light blue) EBNA2 binding sites were compared for CBF1 and EBF1 enrichment in LCLs. The average signal intensities for all EBF1 and all CBF1 peaks in LCLs are shown as reference for comparison (green), respectively. (C) The underlying data of panel B was used to generate box plots depicting signal distributions. An unpaired two-tailed t-test with Welch’s correction (**** p < 0.0001) was performed to determine significant differences between means. Box plot whiskers extend to 1.5x interquartile range. (D) Scatter plots of CBF1, PU.1, and EBF1 versus EBNA2 signal intensities for EBNA2 peaks in LCLs. For each transcription factor the maximal signal intensity was set to 1 to plot signal intensities as relative signal. Each dot represents one EBNA2 peak. Correlation analyses were performed and Spearman correlation coefficients (r_s_) were calculated for each pair. A perfect correlation results in a line (upper left panel) and r_s_ = 1 for EBNA2. Spearman correlation coefficients (rs) were calculated for E2 (1.0), CBF1 (0.46), PU.1/SPI1 (0.19) and EBF1 (0.4). (E) Genome wide quantitative correlation study of EBNA2, CBF1, PU.1, and EBF1 binding intensities represented as matrix. The human genome was divided in 100 bp bins and mapped reads per bin were counted. A correlation coefficient using Spearman correlation was calculated for each TF pair and is displayed and color coded in the matrix.

To further investigate CBF1 independent EBNA2 binding to chromatin, *de novo* motif enrichment analyses of the two subclasses of EBNA2 binding sites were performed separately. Strikingly, the motif of EBF1, an important player in B cell development, was identified as the only and also highly enriched TF motif in the CBF1 independent EBNA2 peak subset, while CBF1 and EBF1 motifs as well as a CBF1/EBF1 composite core motif, show up in the top five motifs of the CBF1 dependent EBNA2 peak set (Fig 5A). In order to look at peak sets of similar size, 243 out of 1546 CBF1 dependent peaks were randomly selected and re-analyzed. For this reduced set, only the CBF1 and EBF1 motifs were significantly enriched. Since the majority of EBNA2 binding sites are also present in LCLs, we could use publicly available ChIP-seq data for EBF1 in LCLs to investigate EBF1 enrichment at CBF1 independent compared to dependent sites (Fig 5B, 5C and S8 Fig). Average CBF1 signal enrichment at EBNA2 binding sites did not significantly differ between CBF1 independent and dependent sites. However, the EBF1 signal was highly and significantly enriched at CBF1 independent compared to CBF1 dependent sites, indicating a potential role for EBF1 in mediating CBF1 independent EBNA2 binding to chromatin. Further quantitative correlation analyses focusing on signal intensities of EBNA2, CBF1, EBF1, and PU.1/SPI1 (Fig 5D and 5E) were performed to rank these co-occurring factors in a quantitative manner. PU.1/SPI1 was included since it had been suggested to serve as a DNA anchor for EBNA2 in the past. As expected, CBF1 showed the highest correlation in signal distribution with EBNA2 at EBNA2 peaks (r_s_ = 0.46) as well as genome wide (r_s_ = 0.5). Most strikingly, EBF1 highly correlated with EBNA2 signals at EBNA2 peaks (r_s_ = 0.4) as well as genome wide (r_s_ = 0.42). However, PU.1/SPI1 and EBNA2 signal intensities correlated weakly at EBNA2 peaks (r_s_ = 0.19) as well as genome wide (r_s_ = 0.17).

### The N-terminal EBNA2 (END) domain of EBNA2 is sufficient to bind EBF1

**Fig 6:**
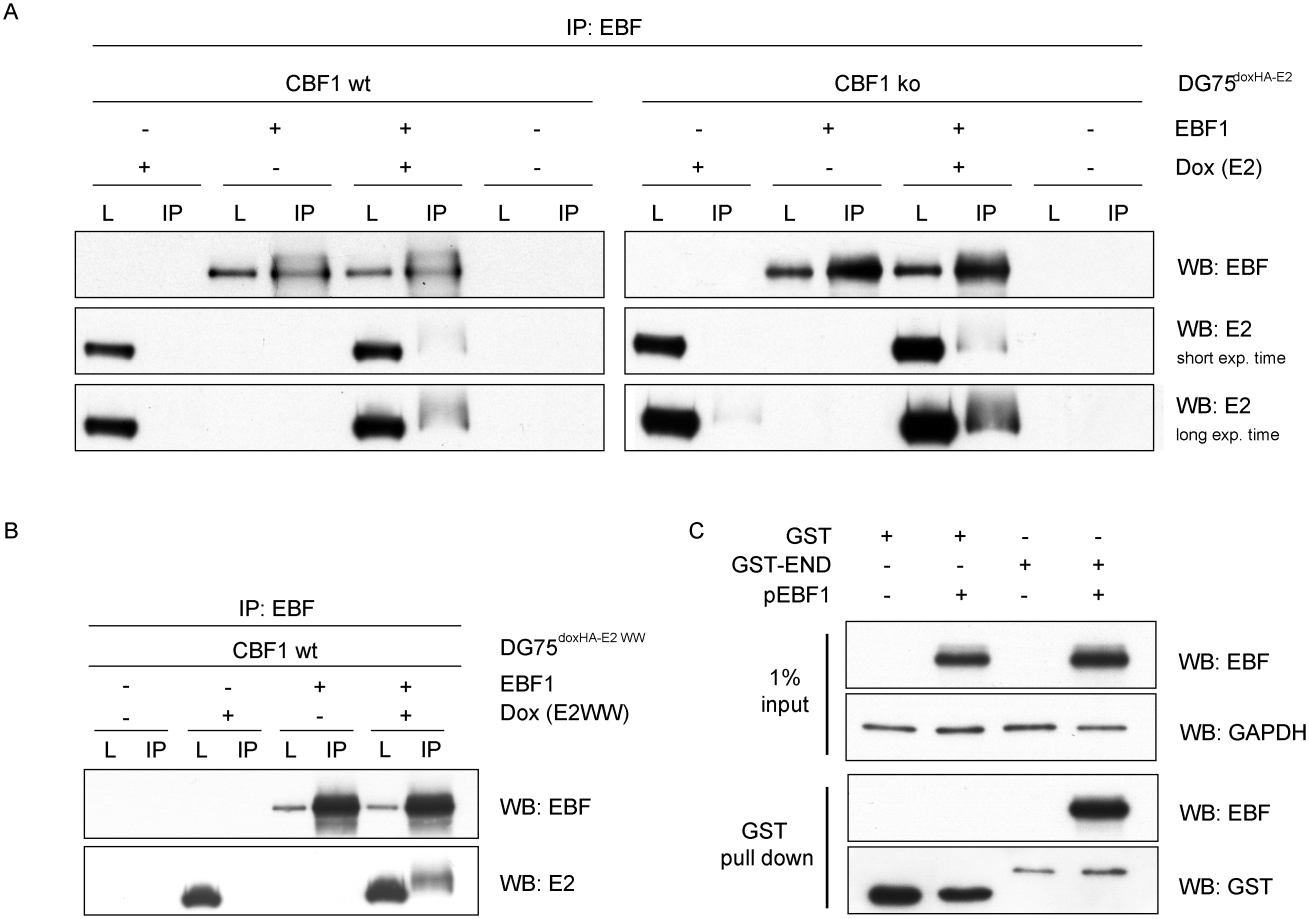
EBNA2 and EBF1 form protein complexes in CBF1 proficient and deficient DG75 B cells. (A) DG75^doxHA-E2^ CBF1 wt and CBF1 ko B cells or (B) DG75^doxHA-E2WW^ CBF1 wt B cells were transfected with EBF1 expression plasmids or empty vector controls. EBNA2 expression was induced by doxycycline (Dox) treatment directly after transfection or cells were left untreated. Total cellular extracts were harvested after 24 h and subjected to immunoprecipitation (IP) using an EBF specific antibody and then assayed by Western blot (WB) using EBF and EBNA2 specific antibodies. Total cell lysates (L) represent 1% of the cells used for IP (n = 2, one representative experiment is shown). (C) DG75 were transfected with EBF1 expression plasmids (+) or empty vector controls (-). Total cellular extracts were harvested 24 h post transfection, incubated with GST or GST-END domain fusion proteins coupled beads to pull down associated proteins. Western blot detection was performed with EBF and GST specific antibodies. GAPDH was used as an internal loading control (n = 2, one representative experiment is displayed).

To test, if EBF1 can bind EBNA2, we performed co-immunoprecipitation (Co-IP) studies in DG75^doxHA-E2^ CBF1 wt and ko cells. After ectopic expression of EBF1 in DG75, an EBF specific antibody co-immunoprecipitated EBNA2 from cellular extracts in both CBF1 proficient as well as CBF1 deficient cells. This indicates that, CBF1 is not required for complex formation of EBF1 with EBNA2 (Fig 6A). The interaction of EBNA2 and CBF1 is well characterized. Two tryptophan residues (WW) within conserved region 6 are absolutely critical for EBNA2 to bind to CBF1 [4]. In order to test, whether the same region might also confer EBF1 binding, we generated DG75 cells expressing an EBNA2 WW325FF mutant (DG75^doxHA-E2-WW^). EBF1 was readily co-precipitated with EBNA2 WW325FF suggesting that EBF1 and CBF1 might target different regions of EBNA2 (Fig 6B).

The N-terminal region of EBNA2, comprising residues 1–58, appears to mediate multiple molecular functions including self-association and transactivation [31, 32]. We have recently described the three-dimensional structure of the EBNA-2 N-terminal dimerization (END) domain by heteronuclear NMR-spectroscopy. The END domain monomer comprises a small fold of four β-strands and an α-helix which form a parallel dimer by interaction of two β-strands from each protomer [33]. To further delineate the EBNA2 region that mediates the interaction with EBF1, we expressed glutathione S-transferase (GST) -END domain fusion proteins in bacteria and used the purified recombinant GST-END proteins as baits to affinity capture EBF1. GST-END specifically pulled down EBF1 in EBF1 transfected cells proving that the N-terminal domain of EBNA2 is sufficient to bind to EBF1 (Fig 6C).

### EBF1 recruits EBNA2 to CBF1 independent binding sites

**Fig 7:**
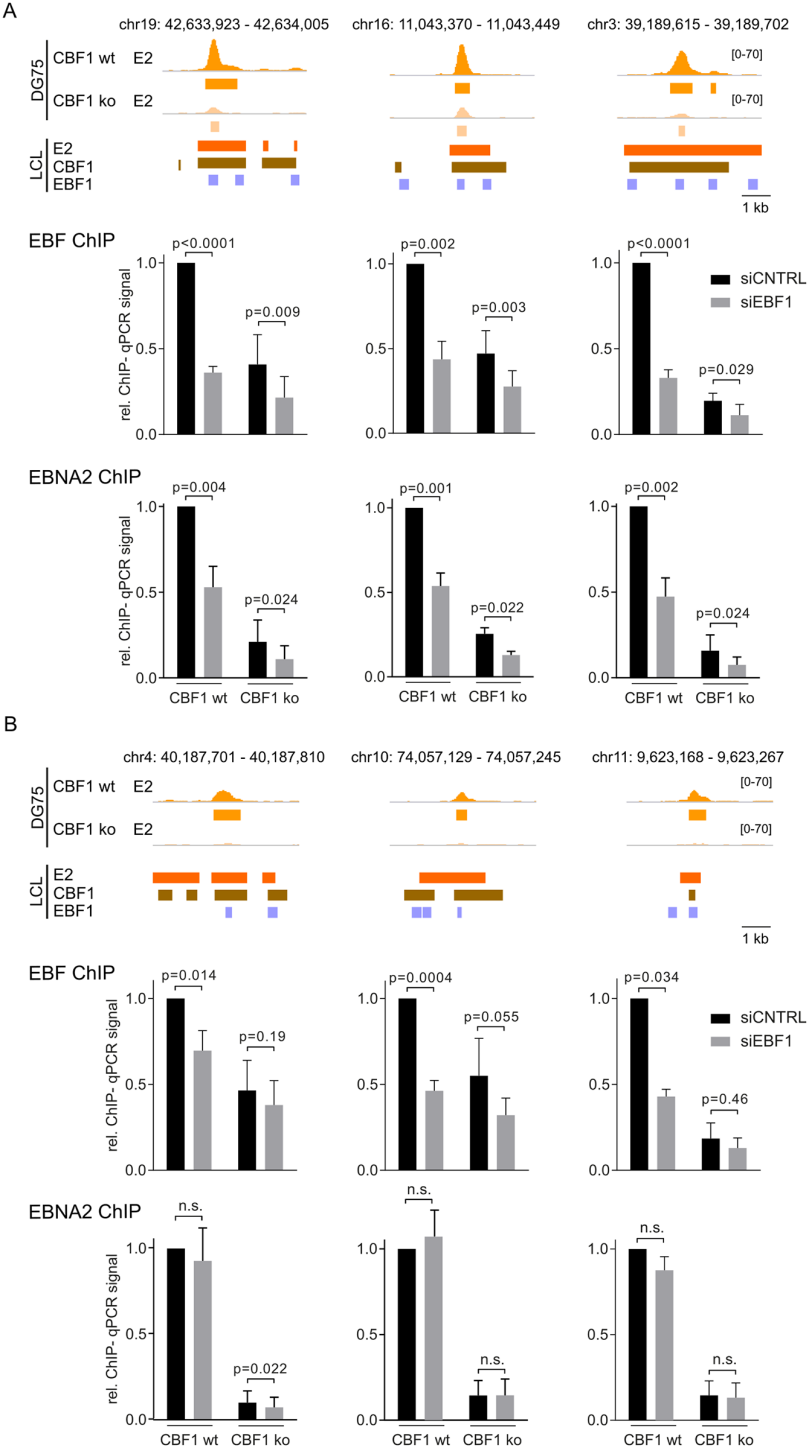
EBNA2 requires EBF1 to bind to its CBF1 independent binding sites. DG75^doxHA-E2^ CBF1 wt or CBF1 ko B cells were transfected with a mixture of scrambled non-targeting siRNAs (siCNTRL) or EBF1 specific siRNAs (siEBF1). 8 h post transfection, EBNA2 transcription was induced. 24 h post transfection, cells were harvested and analyzed by immunoblots (S9 Fig) and ChIP-qPCR. In the upper panel, EBNA2 (E2) binding signal and peak tracks as obtained in DG75^doxHA-E2^ (DG75) as well as EBNA2, CBF1 and EBF1 peak tracks in LCLs are shown for three (A) CBF1 independent or (B) CBF1 dependent EBNA2 binding sites. ChIP-qPCR results for EBF (middle panel) and EBNA2 (lower panel) binding to chromatin before and after EBF1 knock down are shown. Data are mean values, whiskers display standard deviations, p-values, based on a two tailed, paired t-test, are indicated.

Since CBF1 was neither required nor inhibitory for EBF1/EBNA2 complex formation, we asked if EBNA2 needs EBF1 to bind to either CBF1 independent or dependent chromatin sites. To this end, EBF1 protein levels were strongly reduced by siRNA mediated knock down (S9 Fig). EBF1 and EBNA2 binding to chromatin was tested by ChIP followed by quantitative PCR (ChIP-qPCR) for six selected enhancer loci, three CBF1 independent and three CBF1 dependent (Fig 7) sites, which also bind CBF1 and EBF1 in LCLs. While EBNA2 binding to CBF1 independent peaks was significantly reduced after EBF1 knock-down, CBF1 dependent EBNA2 binding was not significantly changed at reduced EBF1 levels. Thus, although EBF1 can bind to CBF1 dependent peaks it does not contribute to EBNA2 recruitment in this context.

## Discussion

### EBNA2 can regulate cellular gene expression in CBF1 deficient B cells

Despite the ubiquitous expression of its anchor protein CBF1, EBNA2 is preferentially recruited to B cell specific enhancers and super enhancers [10, 11, 20, 34, 35]. The underlying mechanism that recruits EBNA2 specifically to these sites in B cells is still not understood and hard to study in the constitutive presence of CBF1. Since it was expected and also shown by other labs that CBF1 knock-down is not compatible with long term proliferation of LCLs [16, 17], we used a CBF1 deficient EBV negative B cell line to study whether EBNA2 can activate cellular genes and bind to chromatin in the absence of CBF1. This CBF1 deficient B cell line had been generated by targeted homologous recombination in DG75, a somatic cell line derived from an EBV negative Burkitt's lymphoma [36]. The proliferation of DG75 cells is driven by the reciprocal t (8;14) translocation which hyper-activates c-MYC expression and which renders proliferation of this cell line CBF1 independent [21].

EBNA2 target gene expression has been intensively studied but due to the omnipresence of CBF1, CBF1 independent target genes, direct or indirect, have never been discovered [21, 22, 37–46]. Unfortunately, a direct comparison of target gene lists across all studies and biological systems that have been published would be misleading, since different methodologies, thresholds and time points were applied. In addition, the use of different gene array systems does not allow the re-analysis of primary data sets as we have done for ChIP-Seq results taken from published data sets. Under these circumstances, the absence of evidence does not provide evidence for absence.

The comparison, however, can be made for selected EBNA2 target genes which were analyzed under similar conditions. While some target genes were identified in all studies, others appear specifically in distinct B cell lines as exemplified by the EBNA2 target gene CXCR7 which is induced in LCLs and BL41, a Burkitt's lymphoma cell line, but not in BJAB, a human lymphoblastoid B cell line [22, 46]. These findings suggest that activation of a subset of EBNA2 target genes requires specific cellular factors which, unlike CBF1, are not ubiquitously expressed. The DG75 cell lines used here express extremely low levels of the cellular transcription factors IRF4 and BATF, which are both well expressed in LCLs (S7C Fig) and highly enriched at LCL unique EBNA2 binding sites (Fig 2D). In addition, chromatin signatures at enhancer positions that can be bound by EBNA2 are distinct for DG75 and naïve B cells (Fig 2C). Thus, EBNA2 target gene activation is fine-tuned by multiple factors in B cells. We expect that additional rate limiting transcription factors apart from EBF1 control EBNA2 functions. A comparative analysis of CBF1 proficient and deficient B cells with distinct transcription factor signatures will be required to identify these additional factors.

Our genome wide gene expression studies confirm previously described EBNA2 cellular target genes which are also induced in LCLs like CD21, SLAMF1, RHOH, HEY1 or CCR7 [22] and in addition identify novel cellular EBNA2 target genes including long non-coding RNAs and micro RNAs. Notably, EBNA2 also controls a smaller but well defined set of CBF1 independent target genes. A selection of targets was validated by qPCR and confirmed the robust regulation of targets in both cell lines proving a strong biological activity of EBNA2 in CBF1 deficient B cells.

It is important to note that EBNA2 not only activates a set of direct target genes but thereby initiates a cascade of secondary events, which are included in our target gene lists and in total reflect EBNA2 functions. CBF1 dependent induced targets were strongly enriched for biological processes involved in immunoglobulin receptor binding functions and a broad array of enzymatic activities. While CBF1 independent EBNA2 induced targets were not significantly enriched for any biological processes, repressed and CBF1 independent targets could be assigned to multiple biological processes involving immune responses. Some of these repressed B cell specific genes like CD79A/mb1, CD79B/B29, VpreB3 have been described previously [21, 22, 47]. These targets are well characterized EBF1 induced target genes in mice [19, 48–51] and have been confirmed in human cells [52]. Recently, it has been demonstrated that EBNA2 promotes the formation of new CBF1 and EBF1 chromatin binding sites [17]. We speculate that EBNA2 might redirect EBF1 to novel chromatin sites and thereby deplete EBF1 activities required for target gene activation.

### EBF1 is a chromatin anchor for EBNA2 at CBF1 independent EBNA2 binding sites

Several lines of evidence support a dynamic model for CBF1/DNA complex formation. Rather than functioning as a pre-bound DNA anchor, this dynamic model suggests that CBF1 is recruited to its DNA binding sites when complexed to cellular or viral binding partners. Notch [53], EBNA2 [17, 44], the EBV viral protein EBNA3C [54] and also RTA [55], the KSHV derived CBF1 binding protein, all promote CBF1/chromatin complex formation and influence chromatin site recognition. We propose that additional tissue-specific cellular or viral factors guide CBF1 associated activator or repressor proteins to functional regulatory elements in the cell.

Our genome-wide EBNA2 ChIP-Seq studies revealed that EBNA2 can bind to chromatin in a CBF1 independent manner. We used publicly available information on transcription factor occupancy in LCLs or peripheral human B cells to characterize different subpopulations of EBNA2 binding sites: i) EBNA2 binding sites shared by or unique to either LCLs or DG75 and ii) CBF1 independent and dependent binding sites. The total number of EBNA2 binding sites found in DG75 cells was significantly smaller than the number of binding sites found in LCLs, although EBNA2 was expressed abundantly in DG75 transfectants. Most EBNA2 binding sites initially identified in DG75 cells were shared with LCLs (S8 Fig). In LCLs, CBF1 independent binding sites score as strong EBNA2 binding sites (Fig 3D and 3E) and EBF1 is significantly enriched (Fig 5B and 5C).

*In silico* transcription factor binding analysis predicted CBF1, EBF1 and MEF2 to be bound at CBF1 dependent binding sites while CBF1 independent EBNA2 binding sites were predicted to bind EBF1 only. Thus these latter binding sites might have low affinity for CBF1 suggesting that EBF1 might be a B cell specific chromatin co-factor for EBNA2, which enhances complex formation also in CBF1 proficient LCLs and DG75 at sites with low affinity for CBF1. Interestingly, we observed that EBNA2 can enhance EBF1 expression and thus might further support complex formation (S1 Table).

For our study, we re-analyzed publicly available primary data sets and correlated signal intensities of transcription factors either at a genome wide level or by focusing on EBNA2 binding sites. These quantitative correlation studies on CBF1, PU.1/SPI1, EBF1, and EBNA2 signal intensities revealed a strong positive correlation of CBF1 and EBF1 to EBNA2 and weak correlation of CBF1 and EBF1 to each other. Surprisingly, PU.1/SPI1 binding activity correlated with neither EBNA2, nor CBF1 nor EBF1 binding activity (Fig 5D and 5E). A physical interaction of PU.1/SPI1 and EBNA2 has been described, but was never characterized in detail [56, 57]. Transient promoter reporter studies had previously suggested that both, PU.1/SPI1 and CBF1 are critical for transactivation of the viral LMP1 promoter by EBNA2 [13, 15, 58]. However, inactivation of the PU.1/SPI1 binding site at the LMP1 promoter in the viral genome did not grossly change the transformation potential of the viral mutants. LMP1 expression and proliferation was diminished but not abolished while inactivation of the EBF1 binding site ablated LMP1 expression [16]. To date, there is no experimental proof indicating that EBNA2 is recruited to chromatin by PU.1/SPI1 [17]. If the pioneer factor PU.1/SPI1 does not serve as chromatin anchor for EBNA2, it could facilitate the access of transcription factors to compacted chromatin or prevent chromatin silencing at the respective enhancer regions [59].

In order to define the contribution of EBF1 to EBNA2 chromatin binding, EBF1 protein expression was downregulated by siRNA. These knock down experiments proved that EBNA2 needs EBF1 to bind efficiently to CBF1 independent chromatin sites in both, CBF1 proficient and deficient cells. In contrast, EBNA2 binding to CBF1 dependent sites was not impaired by EBF1 siRNA knock down and thus was defined to be EBF1 independent although EBF1 is present (Fig 7B). EBF1 and EBNA2 binding is consistently weaker in CBF1 deficient compared to CBF1 proficient DG75 cells (Fig 7). Surprisingly, EBF1 binding is elevated at CBF1 independent sites in CBF1 proficient LCLs (Fig 5). Thus, CBF1 might contribute to the assembly of EBNA2/EBF1 complexes on chromatin, a concept which is consistent with findings of Lu et al. (16), describing EBF1/CBF1 co-occupied binding sites which are preferentially formed in the presence of EBNA2.

### EBF1 and CBF1 bind to different regions of EBNA2

Here we show that EBNA2 and EBF1 can form complexes in cells and thus provide the first evidence that EBF1 interacts with a viral protein. Only a few cellular binding partners of EBF1 have been described so far. EBF1 can bind DNA as a homodimer [60], but can further interact and cooperate with other transcription factors like MEF2C [61], the deoxygenase TET2, an enzyme involved in the DNA demethylation process [62], or the histone acetyltransferase CBP [63]. EBF1 also binds to CNOT3, a subunit of the CCR4-NOT complex [64] which regulates multiple steps in RNA metabolism including transcription, nuclear RNA export and RNA decay [65], and thereby also modulates target gene profiles of EBF. In addition, two multi-zinc finger proteins, ZNF423 and ZNF521, antagonize the biological activity of EBF1 and thereby might promote tumorigenesis [66]. It should be mentioned that in B cells, with a single exception (CNOT3), these interactions have been described after expressing at least one binding partner ectopically or using cross-linking reagents before co-immunoprecipitations have been performed [61]. Thus, it appears that EBF1 protein-protein interactions are particularly difficult to detect at endogenous expression levels in B cells. To date we and others have tried and failed to detect EBNA2/EBF1 complexes expressed at endogenous levels in LCLs while EBNA2/CBF1 complexes could be readily detected in LCLs [17]. Here we detect EBF1/EBNA2 complexes after overexpression of both binding partners in cells. Importantly, the purified END domain consisting of 58 amino acids of EBNA2 is sufficient to specifically affinity capture EBF1 from cellular extracts.

Future studies on purified proteins of both binding partners will reveal whether the interaction of EBNA2 and EBF1 is direct or whether so far unknown factors, proteins or DNA, support complex formation. If additional cellular factors promote complex formation, they need to be expressed at very high levels in the cell to efficiently bridge viral and cellular proteins.

In summary, the genetic ablation of CBF1 expression in B cells provides novel valuable insights into the molecular mechanisms of EBNA2 activity. At this point of our study, we can define EBNA2 functions in the absence of CBF1. Chromatin conformation capture techniques performed in DG75 cells will be required to link EBNA2 binding sites to the respective target genes. Since EBNA2/EBF1 complex formation could be demonstrated in CBF1 proficient and deficient cells and EBF1 and CBF1 bind to different regions of EBNA2, heterotrimeric complexes might be formed. Whether these complexes activate or repress transcription might depend on their composition and the chromatin context of enhancer and promoters they bind to. Any working hypothesis to be tested will have to take into account the dimeric nature of EBNA2 and EBF1 as well as the fact that CBF1 and EBF1 are co-expressed and their binding motifs might overlap [67]. Our future studies will need to explore the architecture of these complexes in order to understand if pre-formed EBNA2/CBF1 complexes can use EBF1 to guide EBNA2 to B cell specific enhancers and thereby provide B cell specificity to EBNA2 activities.

## Materials and methods

### Plasmids

*pcDNA3* (pCDNA3) and EBF1-myc expression plasmid (pCDNA3.EBF1-5xmyc) were kindly provided by Mikael Sigvardsson [68]. pCKR74.2 is a Dox (doxycycline) inducible HA- (haemagglutinin) tagged EBNA2 expression plasmid (pCKR74.2) based on pRTR [69, 70].

### Cell lines and cell culture conditions

The cells were maintained as suspension cultures in RPMI 1640 medium (Gibco Life Technologies) supplemented with 10% FCS (fetal calf serum, Bio&Sell), 4 mM L-Glutamine and 1 x penicillin/streptomycin (Gibco Life Technologies). 721 is an EBV positive LCL cell line [71]. The DG75 ko cell line (SM224.9), DG75^ER/EBNA2^ CBF1 wt and ko cells (SM295 and SM296) have been described before [21, 22]. The ER/EBNA2 (estrogen receptor hormone binding domain EBNA2) fusion protein was activated by cultivating the cells in cell culture medium supplemented with 1 μM ß-estradiol. The DG75^doxHA-E2^/CBF1 wt (CKR128-34) and the DG75^doxHA-E2^/CBF1 ko (CKR178-10) cell lines carry the Dox inducible HA-EBNA2 expression plasmid (pCKR74.2). DG75^doxHA-E2WW^/CBF1 wt (CKR436) expresses a Dox inducible HA-EBNA2 WW325FF mutant (pCKR421). They were cultivated in 1 μg/ml puromycin containing media. EBNA2 expression was induced by doxycycline treatment (1μg/ml).

### Genome wide expression analysis by application of the Human Gene 2.0 ST array (Affymetrix) and relative quantification of transcripts by real-time RT-PCR

Total RNA was extracted from 1x10^7^ cells induced for 24 h with 1 μM ß-estradiol using the Qiagen RNeasy Mini Kit. Expression analysis starting from 100 ng of total cellular RNA was performed using the Ambion WT Expression Kit (Applied Biosystems) and subsequently the GeneChip WT Terminal Labeling and Hybridization Kit (Affymetrix) followed by the GeneChip Human Gene 2.0 ST array (Affymetrix) according to the manufacturer's protocol. All affymetrix CEL files have been processed in Bioconductor/R using robust multiarray average (RMA) for normalization and summarization and limma for differential expression and significance. Quality has been checked using the array QualityMetrics package. Additional filtering based on the fold change between the two conditions was applied with different stringency, individually described in the legend of the tables and figures. Analyzation and visualization of the Microarray was performed using Genesis, available at http://genome.tugraz.at. Quantitative RT-PCR analysis was performed as described previously [72]. Primers used for RT-qPCR were designed applying Primer3 software (http://primer3.ut.ee/) and selection of mature transcripts was ensured by amplification across exon-exon junctions. Primers used for quantitative RT-PCR are summarized in S3 Table. All data were normalized for the relative abundance of the Actin B transcript.

### Gene ontology analysis

GOrilla is a tool to identify and visualize enriched GO terms in ranked lists of genes (http://cbl-gorilla.cs.technion.ac.il/) [73]. Enrichment is defined as E = (b/n) / (B/N), with N = the total number of genes, B = the total number of genes associated with a specific GO term, n = the number of genes in the top of the user's input list and b = the number of genes in the intersection. The threshold for n is selected by GOrilla by maximizing E and statistical significance is computed taking into account the multiple hypothesis tests arising due to the maximization.

All GO terms for which B < 10 were ignored. GO terms with a q-value (FDR) ≤ 10^−4^ were selected and ranked for their enrichment score given by GOrilla.

As induction and repression was stronger in DG75ER/EBNA2 CBF1 wt cells than in CBF1 ko cells, principal component analysis (PCA) was used to identify genes regulated on average or differentially between wt and ko (S6 Fig). PCA was performed for all genes significantly regulated in CBF1 wt or ko cells (limma q < 0.01). The first principal component corresponded to average regulation while the second principal component represented CBF1 dependence. Genes were first ranked according to the first principal component, i.e. top entries corresponded to genes that were induced on average in CBF1 wt and ko cells. This was repeated after reversing the list to analyze genes repressed on average. Furthermore, from each of these two lists, the top 2000 genes were selected and both were ranked according to the second principal component. Both lists were additionally reversed. Therefore, in these four additional lists, genes that are either induced or repressed on average were ranked according to their degree of CBF1 dependence.

### Immunoprecipitation (IP)

1x10^7^ DG75^doxHA-E2^/CBF1 wt or ko cells were lysed in 500 μl NP-40 lysis buffer (1% NP-40, 150 mM NaCl, 10 mM Tris-HCL pH 7.4, 1mM EDTA pH 8.0, 3% Glycerol) supplemented with complete protease inhibitor cocktail (Roche) for 1h (30 min rolling at 4°C, 30 min on ice). Precleared protein lysates were used for co-immunoprecipitation by adding 100 μl of hybridoma supernatant (E2: α-HA R1 3F10; E.Kremmer) or 1 μg of purified antibody (α-EBF Santa Cruz Biotechnology, sc-137065) at 4°C under rotation overnight. Subsequently, 50 μl of 50% suspension of pre-blocked, equilibrated protein G-coupled Sepharose beads (GE Healthcare) were added to the lysates and incubated for 2h at 4°C under rotation. Immunoprecipitates were washed 5 times with NP-40 lysis buffer, Laemmli buffer was added to the beads, and the samples were boiled, submitted to electrophoresis by SDS-PAGE and analyzed by immunoblotting.

### Immunoblotting (Western blot)

5x 10^6^ cells were lysed in 200 μl NP-40 lysis buffer (1% NP-40, 150 mM NaCl, 10 mM Tris-HCL pH 7.4, 1mM EDTA pH 8.0, 3% Glycerol) for 2 h on ice. 30 μg of total cell lysate were submitted to SDS-PAGE under reducing conditions. Immunoblotting was performed on polyvinylidene difluoride (PVDF) membranes. Western blots were probed with the following primary antibodies: rat α-EBNA2 (R3; IgG2A; E. Kremmer), rat α-CBF1 (RBP-J 7A11, E. Kremmer), rat α-GST (GST 6G9, IgG2A, E. Kremmer), mouse α-EBF (Santa Cruz Biotechnology, sc-137065), goat α-BATF (B-ATF H-19, Santa Cruz Biotechnology, sc-15280), rabbit αIRF4 (IRF4H-140), and-GAPDH (EMD Millipore MAB374). HRP-coupled secondary antibodies (Santa Cruz Biotechnology) and an ECL kit (GE Healthcare) were used for visualization. For subsequent quantification of protein levels, exposed films were scanned in transmission mode and protein band intensities were determined by densitometry using *ImageJ* software (http://rsbweb.nih.gov/ij/) [74].

### Transfection

5x 10^6^ DG75 cells were transfected by electroporation at 250 V and 950 μF in 250 μl reduced serum media (Opti-MEM, Gibco Life Technologies; without supplements) using 0.4 cm-electrode-gap cuvettes (Bio-Rad) and the Bio-Rad Gene Pulser.

### siRNA knockdown in DG75 cells

5x 10^6^ cells were transfected with 100 pmol control siRNA-A or EBF1 siRNA (both Santa Cruz Biotechnology, sc-37007 and sc-10695) by electroporation. 24 h after transfection, 1x 10^7^ induced, siRNA treated cells were harvested for chromatin isolation and 5x10^6^ cells for protein isolation.

### Chromatin immunoprecipitation

This ChIP protocol is based on reference (59) with minor modifications as indicated below. In brief, 2x 10^7^ DG75^doxHA-E2^ cells were harvested and washed twice in ice cold PBS, resuspended in 20 ml RPMI 1640 (Gibco Life Technologies) and formaldehyde (1% final) was added for cross-linking. The reaction was stopped by addition of glycine (125 mM final) after 7 min and gentle shaking for 5 min at RT. Cells were pelleted and washed twice in ice cold PBS. Nuclei were isolated by washing the cells 3x with 10 ml of ice cold Lysis Buffer (10 mM Tris-HCl, pH 7.5, 10 mM NaCl, 3 mM MgCl2, 0.5% NP-40, 1x proteinase inhibitor cocktail (PIC, Roche)) and subsequent centrifugation (300 g for 10 min at 4°C). Nuclei were resuspended in 1 ml Sonication Buffer (50 mM Tris-HCl, pH 8.0, 10 mM EDTA, pH 8.0, 0.5% SDS, 1x PIC) and incubated on ice for 10 min. Chromatin was sheared to an average size of 200–300 bp by four rounds of sonication for 10 min (30 sec pulse, 30 sec pause) using a Bioruptor device (Biogenode). Cell debris was separated by centrifugation at maximum speed for 10 min at 4°C and chromatin containing supernatants were stored at -80°C or directly used for IP. To prepare input DNA, 25 μl aliquots (1/10 of the amount used per IP) were saved at -80°C. For IPs 250 μl chromatin (equals 5x 10^6^ cells) were diluted 1:4 with IP Dilution Buffer (12.5 mM Tri-HCl, pH 8.0, 212.5 mM NaCl, 1.25% Triton X-100, 1 x PIC) and incubated with 100 μl of hybridoma supernatant on a rotating platform at 4°C overnight. A combination of EBNA2 and HA-tag specific antibodies (⅓ α-E2 R3 (rat IgG2a, ⅓ α-E2 1E6 (rat IgG2a), and ⅓ α-HA R1-3F10 (rat IgG1)) was used to precipitate EBNA2 and an isotype-matched unspecific antibody mixture (⅔ α- GST 6G9 (rat IgG2a) and ⅓ α-CD23 Dog-CD3 (rat IgG1) both by E. Kremmer) was used as isotype control. The EBF antibody (C-8) (sc-137065, Santa Cruz Biotechnology) was used to precipitate EBF1 and an antibody specific for ovalbumin (M-Ova 3D2, E. Kremmer) was used as an isotype control. Protein G sepharose (GE Healthcare) was equilibrated with IP Dilution Buffer, added to the lysate and incubated at 4°C for 4 h with constant rotation. Beads were extensively washed with: 2x Wash Buffer I (20 mM Tris-HCl, pH 8.0, 2 mM EDTA, pH 8.0, 1% Triton X-100, 150 mM NaCl, 0.1% SDS, 1x PIC), 1x Wash Buffer II (20 mM Tris-HCl, pH 8.0, 2 mM EDTA, pH 8.0, 1% Triton X-100, 500 mM NaCl, 0.1% SDS, 1x PIC), 1x Wash Buffer III (10 mM Tris-HCl, pH 8.0, 1 mM EDTA, pH 8.0, 250 mM LiCl, 1% NP-40, 1% sodium deoxycholate, 1x PIC) for 5 min under rotation, and 2x with TE (10 mM Tris-HCl, pH 8.0, 1 mM EDTA, pH 8.0) for 1 min. Protein-DNA complexes were eluted with 2x 150 μl Elution Buffer (25 mM Tris-HCl, pH 7.5, 10 mM EDTA, pH 8.0, 1% SDS) at 65°C for 15 min. Input samples were adjusted to 300 μl with Elution Buffer. Eluates and input samples were incubated with Proteinase K (1.5 μg/μl final, Roche) for 1 h at 42°C. Cross-linking was reversed by incubation at 65°C overnight. DNA was recovered using QIAquick PCR purification kit (Qiagen).

The EBNA2 specific ChIP in LCL was performed as described above with the following modifications: Protein-protein interactions were fixated by adding disuccinimidyl glutarate (DSG, Pierce #20593, 2 mM final, using freshly prepared 0.5 M stock solution in DMSO) for 23 min at RT and prior to formaldehyde (1% final) cross-link for additional 7 min. Sonication Buffer was composed of 50 mM Tris-HCl, pH 8.0, 5 mM EDTA, pH 8.0, 0.5% SDS, 0.5% Triton X-100, 0.05% sodium deoxycholate, and 1x PIC. IP Dilution Buffer was composed of 12.5 mM Tri-HCl, pH 8.0, 187.5 mM NaCl, 1.25 mM EDTA, pH 8.0, 1.125% Triton X-100, and 1 x PIC. For EBNA2 specific IP 50 μl of α-E2 R3 (rat IgG2a) and 50 μl α-E2 1E6 (rat IgG2a) hybridoma supernatant were applied and the same volume of isotype-matched nonspecific antibody (α- GST 6G9 (rat IgG2a) E. Kremmer) was used as negative control.

### Whole-genome chromatin immunoprecipitation DNA sequencing (ChIP-seq)

For sequencing purposes DNA concentration was measured using the Qubit dsDNA HS Assay Kit (Thermo Fisher). A maximum of 100 ng ChIP or input derived DNA were used for library preparation (NEBNext Ultra DNA Library Prep Kit for Illumina) and subsequently subjected to deep sequencing using a HiSeq 1500 device (Illumina).

### Chromatin immunoprecipitation quantitative polymerase chain reaction (ChIP-qPCR)

The amount of recovered DNA in input samples and after IP with specific antibody or an unspecific isotype-matched IgG control was quantified by qPCR using primers listed in S3 Table.

qPCR was performed using LightCycler 480 SYBR Green I Master (Roche) on a LightCycler 480 II instrument (Roche) as described previously [72]. 2 technical replicates were analyzed for each biological replicate. Amplification was always conducted at 63°C. To account for differences in amplification efficiencies a standard curve was generated for each primer pair using serial dilutions of sheared DNA (input) as template. DNA quantities detected in input samples were adjusted to the amount of chromatin used per IP by multiplication with 20. Values obtained from IP samples with unspecific IgG control were subtracted from the DNA amounts recovered by IP with specific antibody. The percent of input was calculated as (DNA from specific IP corrected for IgG control background/ DNA input) x 100. To validate the ChIP, qPCR at a known (ChIP-Seq) positive locus was performed. To compromise divergent EBNA2 inducibility in wildtype and knockout cells, the percent input was calculated relative to a known negative locus (ChIP-Seq; percent input at tested locus/percent input of known negative locus). To display the change in binding, the mean relative input of the wildtype cells treated with control siRNA was set to one. A paired t-test was performed to assess significance of differences of means.

### Expression and purification of GST fusion proteins

Expression plasmids were transformed into *E*.*coli* strain BL21. Bacteria were cultured in 400 ml of LB medium containing antibiotics at 37°C until an OD of 0.5–0.7 was reached. Expression of proteins was induced with 1 mM IPTG for 3 h at 30°C. After induction, bacteria were suspended in 20 ml ice cold binding buffer (25 mM HEPES, pH 7.6, 0.1 mM EDTA, pH 8, 12.5 mM MgCl_2_, 10% Glycerol, 0.1% NP-40, 100 mM KCl, 1 mM PMSF, 1 mM DTT, 1 mM PMSF) and lysed by sonication. Lysates were cleared by centrifugation at 48,000 x g at 4°C for 20 min. Glutathione Sepharose 4B beads (GE Healthcare) were washed and resuspended in binding buffer to prepare a 50% slurry. To coat the beads with GST or GST fusion protein, 100 μl of the 50% slurry were incubated with the 20 ml of cleared lysates for 1 hat 4°C and washed 3 times with 20 ml binding buffer.

### GST pull down assay

1x10^7^ DG75 cells were transfected with EBF1 expression plasmids or empty vector controls. 24 h after transfection, cells were harvested and lysed in 500 μl lysis buffer (50 mM HEPES, pH 7.6, 5 mM EDTA, pH 8, 150 mM NaCl, 0.1% NP-40, 1 mM PMSF) followed by sonication. Cell lysates were centrifuged for 15 min at 16,000x g, 4°C, and the protein concentration was measured by Bradford assay. To pull down EBF1, the supernatants were incubated with the GST or GST fusion protein coated beads for 3 hat 4°C. Subsequently, beads were washed 5 times with binding buffer and protein complexes were dissolved in 2x Lämmli buffer (4% SDS, 20% Glycerol, 120 mM Tris/HCl, pH 6.8, 5% β-Mercaptoethanol, Bromphenol-blue). Samples were analyzed by SDS-PAGE and Western Blot.

### Bioinformatics

All bioinformatic analyses of ChIP-Seq data were conducted by using the galaxy bioinformatics platform [75] hosted and maintained by the Bioinformatics Department of the University of Freiburg. For all sequenced samples, at least 17 million reads were obtained and biological duplicates of EBNA2 ChIP and input samples were sequenced. Reads were mapped to the human genome using Bowtie2 [76]. For all samples, at least 95% of reads were mappable to the human genome including at least 69% of uniquely mapping reads with one distinct location (S4 Table). Biological duplicates of mapped reads were merged and subsequently significant EBNA2 binding sites were identified using MACS2 [77] by normalizing ChIP to input samples (S4 Table). In a second step, the peaks were further filtered and “negative peaks” (negative amplitude, significantly higher read count in the input sample), peaks located at black-listed regions [78], peaks with a very low enrichment score, and such located on chromosomes not included in the ENCODE data for GM12878 (e.g. chrY, chrUn) were excluded (S4 Table). Normalized EBNA2 ChIP signal tracks were generated by subjecting duplicate-merged ChIP and input read files to bamCompare of the deepTool package [79] and normalizing ChIP to input samples by subtraction as well as normalizing to fragments (reads) per kb per million (RPKM) to account for genome coverage. Mean signal intensities at specific peak sets were calculated using computeMatrix of the deepTools package. This workflow for transcription factor peak calling and signal track generation was applied to all ChIP-seq data sets analyzed in this manuscript. A separate workflow for the analysis of histone modification ChIP-seq data was generated to account for the typical broader signal distribution and applied to all such data sets analyzed in this study. Data provided by public resources were reanalyzed using the same pipeline as described above and references are listed in S5 Table. The details of all analyses steps are captured in a Galaxy workflow which can be downloaded at github (https://github.com/bgruening/galaxytools/tree/master/workflows/peak_calling) and re-run and analyzed in Galaxy. Cluster analysis was performed using the k-means algorithm tool (numbers of clusters expected = 12, max. iterations = 50) provided by Genesis (release 1.7.7), available at http://genome.tugraz.at. Genesis was also used to generate heatmaps [80].

### Flow cytometry

Inducibility of EBNA2 expression in DG75^doxHA-E2^/CBF1 wt and ko cell lines was evaluated by monitoring the expression of the eGFP surrogate marker of pCKR74.2. Cells were induced for 16 h or 24 h with doxycycline, washed and fixed with 0.5% PFA in PBS. For quantification of induced cells, the FACSCalibur system (BD Biosciences) and CellQuest Pro software (BD Biosciences) were applied.

## Supporting information

S1 Fig Control panels documenting estrogen responses in ER/EBNA2 expressing DG75 cells compared to estrogen treated untransfected parental cell lines. (A) DG75 parental cells (DG75 CBF1 wt), CBF1 deficient (DG75 CBF1 ko), ER/EBNA2 expressing (DG75^ER/EBNA2^ CBF1 wt), and CBF1 deficient ER/EBNA2 expressing DG75 cells (DG75^ER/EBNA2^ CBF1 ko) were treated with estrogen for 24 h or were left untreated. Total cellular RNA was isolated and submitted to gene expression analysis using the Human Gene 2.0 ST array. All probe sets represent single transcripts. For each condition 3 biological replicates were examined. Each vertical column in the heatmap represents the results obtained from a single microarray. Horizontal rows represent data obtained for a particular probe set across all cell lines and conditions after normalization of expression values on a scale ranging from -2.0 to 2.0 for each probe set. Expression levels of 950 transcripts which change expression levels at least 2-fold (p ≤ 0.05) in response to estrogen in DG75 ER/EBNA2 cells are displayed. The relative high, medium and low expression values are represented by red, white and blue, respectively. Vertical columns are ranked according to fold changes in ER/EBNA2 expressing DG75 from highest induction on top to highest repression levels at the bottom. (B) RNA expression levels of a panel of previously described estrogen responsive target genes in DG75 cells after estrogen treatment (RMA = robust multi array average). (C) RNA expression level of previously defined EBNA2 target genes in DG75 ER/EBNA2 cells after estrogen induction.
(TIF)

S2 Fig Based on the expression level changes of 950 transcripts which are regulated in DG75^ER/EBNA2^ CBF1 wt at least 2-fold (p ≤ 0.05) and expression levels of the same transcripts in DG75^ER/EBNA2^ CBF1 ko cells, 12 clusters of transcripts were defined. Number of transcripts contained in each cluster is indicated on the left. Unique ID and Gene Name are listed in S2 Table.
(TIF)

S3 Fig Heatmap representing the 132 transcripts regulated at least 2-fold (p ≤ 0.001) by EBNA2 in CBF1 deficient DG75^ER/EBNA2^ cells. Total cellular RNA was isolated and submitted to gene expression analysis using the Human Gene 2.0 ST array. All probe sets represent single transcripts. For each condition 3 biological replicates were examined. Each vertical column represents the results obtained by a single microarray. Horizontal rows represent data obtained for a particular probe set across all cell lines and conditions on a scale ranging from -2.0 to 2.0 for each probe set. The relative high, medium and low expression values are represented by red, white, and blue color, respectively. Vertical columns are ranked according to fold changes in ER/EBNA2 expressing DG75 CBF1 ko from highest induction level on top to highest repression levels at the bottom. The transcript cluster ID and the assigned genes/transcripts are indicated. Note that not more than five assigned genes are listed (*). If no assignment was available the chromosomal position is indicated (**).
(TIF)

S4 Fig Validation of gene array hybridization results by quantitative RT-PCR. (A) Relative transcript levels of EBNA2 target genes were quantified from total RNA samples of the indicated cell lines by RT-qPCR. All results were normalized to actin B transcript levels. (B) For comparison the expression levels measured by gene array hybridization are shown in parallel.
(TIF)

S5 Fig Heatmap showing microRNAs regulated at least 1.5-fold (p ≤ 0.05) by EBNA2 in DG75^ER/EBNA2^ CBF1 wt cells (for all details see S1 Fig). (TIF)

S6 Fig Identification of individual target gene subsets based on principle component analysis. Since on average target gene expression changes in CBF1 positive cells were stronger than in CBF1 negative cells, principle component analysis on EBNA2 regulated genes was used to identify specific subpopulations: The first principle component (green arrow) describes the upregulation of genes in both cell lines, the second principle component (red arrow) describes the degree of CBF1 dependence. The scatter blots depict all genes (A) or the top 2000 (B) induced/repressed genes which are regulated in at least one cell line.
(TIF)

S7 Fig Doxycycline inducible HA-EBNA2 expression in CBF1 proficient or deficient DG75 B cells. (A) pRTRdoxHA-E2 vector used to generate stable DG75 cell lines. The coding sequence for EBNA2 fused to a N-terminal HA-tag (HA-E2), plus a preceding intron of the beta-globin gene for enhanced expression, was cloned into the pRTR vector [69, 70] using SfiI restriction sites. The bidirectional promoter simultaneously drives the expression of HA-EBNA2 and the bicistronic reporter construct consisting of a truncated nerve growth factor receptor gene (tNGFR) and enhanced green fluorescent protein (eGFP) gene upon doxycycline induction. (B) Expression of HA-EBNA2 was induced with 1 μg/ml doxycycline (Dox) for 24 h and monitored by quantifying eGFP expression via flow cytometry and scored at least 89% with a maximum of 5% difference between DG75 CBF1 wt and ko cells. Data from one representative experiment (n = 3) and percentages of induced cells are shown. (C) Western Blot analysis confirming the expression of HA-EBNA2 in DG75doxHA-E2 cell lines 24 h post induction with 1 μg/ml Dox. 721 is an LCL cell line used as positive control for all western blots. The absence of CBF1 expression in the DG75doxHA-E2 CBF1 ko cell line is confirmed. EBF1, IRF4, BATF, and PU.1/SPI1 are shown for comparison. GAPDH serves as loading control. For all blots 30μg protein lysate was used for all lanes with a single exception: For EBNA2 blots 3 μg DG75 lysates and 30μg 721 lysate were used in order to take the 5–10 fold higher EBNA2 expression of DG75 into consideration.
(TIF)

S8 Fig Intersection of EBNA2 binding sites identified in LCLs, DG75doxHA-E2 CBF1 wt or ko. The table lists (lower part) the called peaks and the fraction of peaks which also score in LCL EBNA2.
(TIF)

S9 Fig Validation of efficient EBF1 knockdown by Western blot analysis. DG75^doxHA-E2^ CBF1 wt or CBF1 ko B cells were transfected with a mixture of scrambled non-targeting siRNAs (siCNTRL) or EBF1 specific siRNAs (siEBF1). 8 h post transfection, EBNA2 transcription was induced. 24 h post transfection, cells were harvested and analyzed by immunoblots and ChIP-qPCR. (A) Representative immunoblots showing expression levels of EBNA2, EBF1, CBF1, and GAPDH before and after knockdown (n = 3). EBF1 negative Jurkat cell lysate served as a negative control. (B) Protein band intensities were quantified by densitometry. The change of EBF1 protein expression in siRNA (siEBF1) treated compared to non-treated cells (CNTRL) is significant according to paired t-test when indicated.
(TIF)

S1 Table Gene array (Human Gene 2.0). (XLSX)

S2 Table Gene list k-means clustering (cluster 1–12). (XLSX)

S3 Table Primer qPCR. (PDF)

S4 Table Summary ChIP-seq results. (PDF)

S5 Table Public resources used for this study. (PDF)
