# Supplementary figures and images for: EBF1 binds to EBNA2 and promotes the assembly of EBNA2 chromatin complexes in B cells
Source: PLoS Pathog. 2017 Oct 2;13(10):e1006664. doi: 10.1371/journal.ppat.1006664 (PMC5638620; doi:10.1371/journal.ppat.1006664)

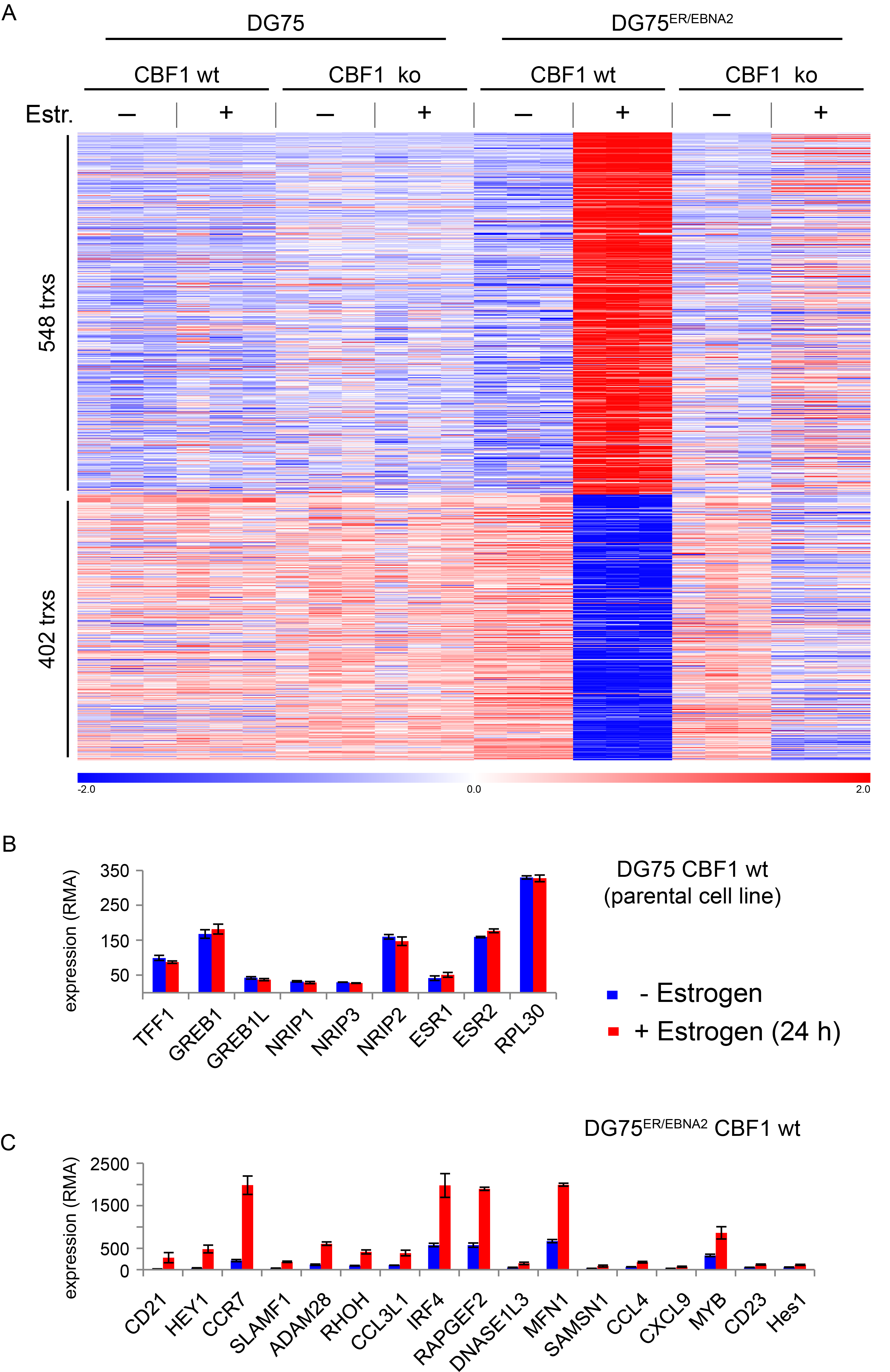

Supplement: S1 Fig — (A) DG75 parental cells (DG75 CBF1 wt), CBF1 deficient (DG75 CBF1 ko), ER/EBNA2 expressing (DG75ER/EBNA2 CBF1 wt), and CBF1 deficient ER/EBNA2 expressing DG75 cells (DG75ER/EBNA2 CBF1 ko) were treated with estrogen for 24 h or were left untreated. Total cellular RNA was isolated and submitted to gene expression analysis using the Human Gene 2.0 ST array. All probe sets represent single transcripts. For each condition 3 biological replicates were examined. Each vertical column in the heatmap represents the results obtained from a single microarray. Horizontal rows represent data obtained for a particular probe set across all cell lines and conditions after normalization of expression values on a scale ranging from -2.0 to 2.0 for each probe set. Expression levels of 950 transcripts which change expression levels at least 2-fold (p ≤ 0.05) in response to estrogen in DG75 ER/EBNA2 cells are displayed. The relative high, medium and low expression values are represented by red, white and blue, respectively. Vertical columns are ranked according to fold changes in ER/EBNA2 expressing DG75 from highest induction on top to highest repression levels at the bottom. (B) RNA expression levels of a panel of previously described estrogen responsive target genes in DG75 cells after estrogen treatment (RMA = robust multi array average). (C) RNA expression level of previously defined EBNA2 target genes in DG75 ER/EBNA2 cells after estrogen induction. (TIF) [file ppat.1006664.s001.tif]

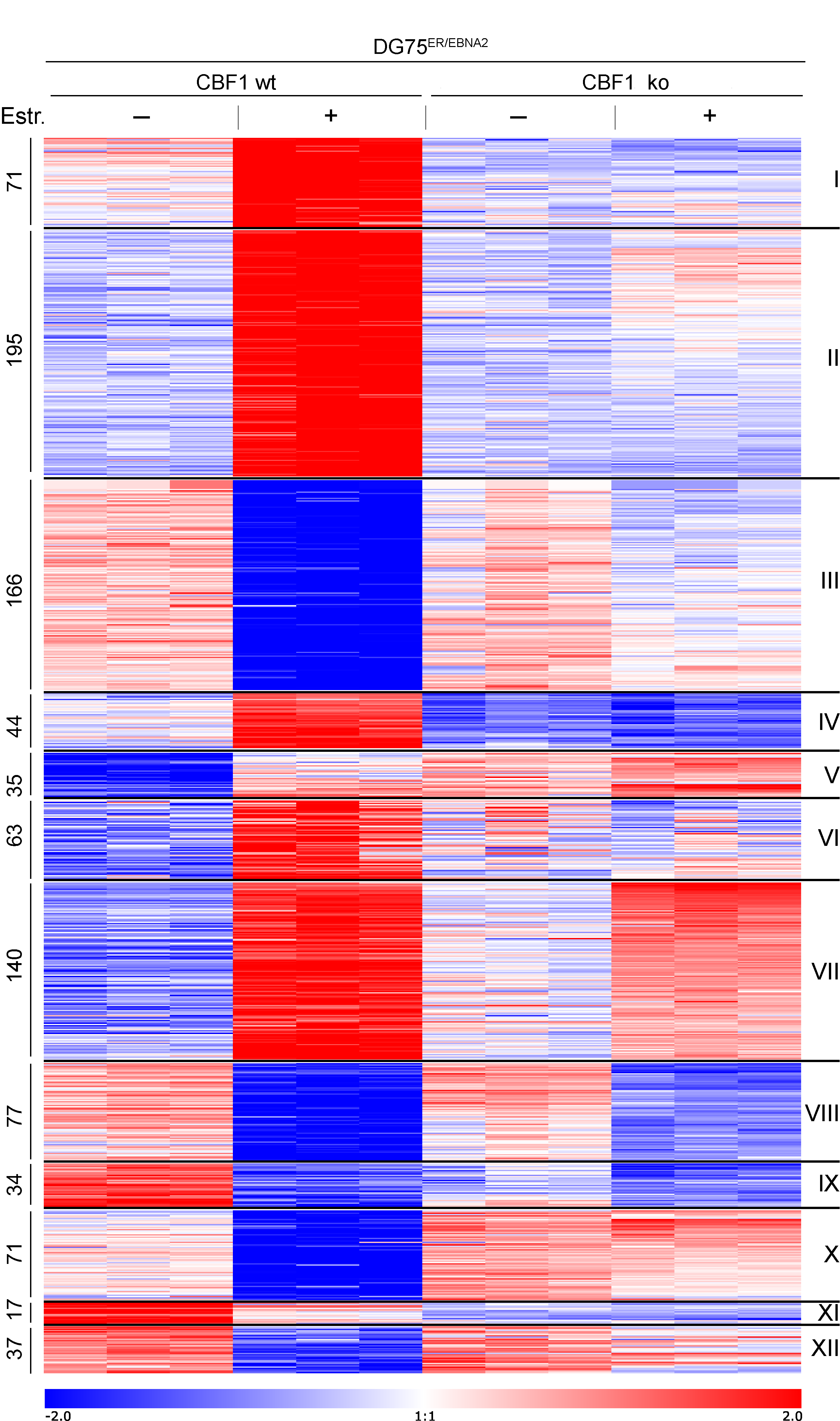

Supplement: S2 Fig — Number of transcripts contained in each cluster is indicated on the left. Unique ID and Gene Name are listed in S2 Table. (TIF) [file ppat.1006664.s002.tif]

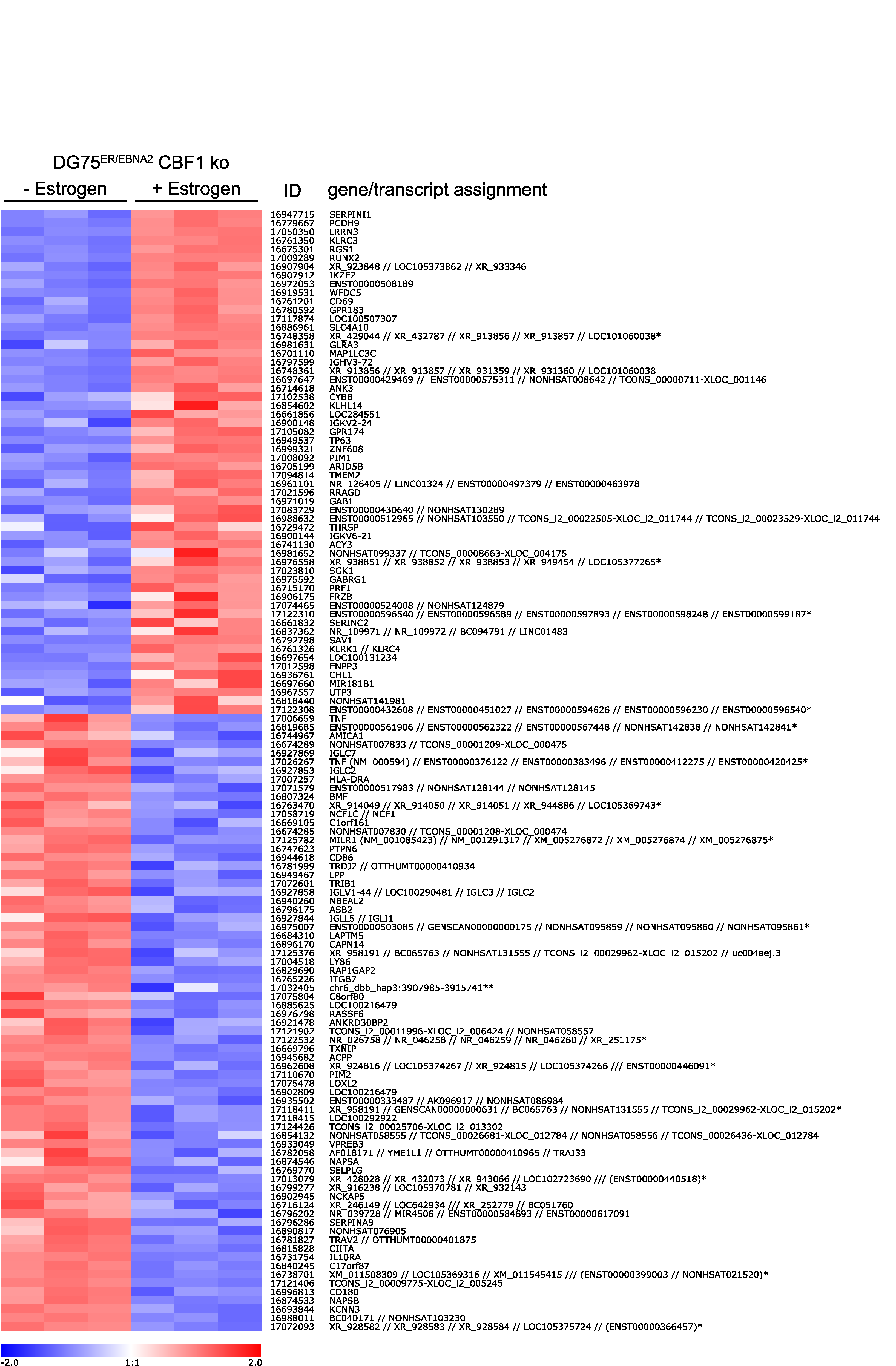

Supplement: S3 Fig — Total cellular RNA was isolated and submitted to gene expression analysis using the Human Gene 2.0 ST array. All probe sets represent single transcripts. For each condition 3 biological replicates were examined. Each vertical column represents the results obtained by a single microarray. Horizontal rows represent data obtained for a particular probe set across all cell lines and conditions on a scale ranging from -2.0 to 2.0 for each probe set. The relative high, medium and low expression values are represented by red, white, and blue color, respectively. Vertical columns are ranked according to fold changes in ER/EBNA2 expressing DG75 CBF1 ko from highest induction level on top to highest repression levels at the bottom. The transcript cluster ID and the assigned genes/transcripts are indicated. Note that not more than five assigned genes are listed (*). If no assignment was available the chromosomal position is indicated (**). (TIF) [file ppat.1006664.s003.tif]

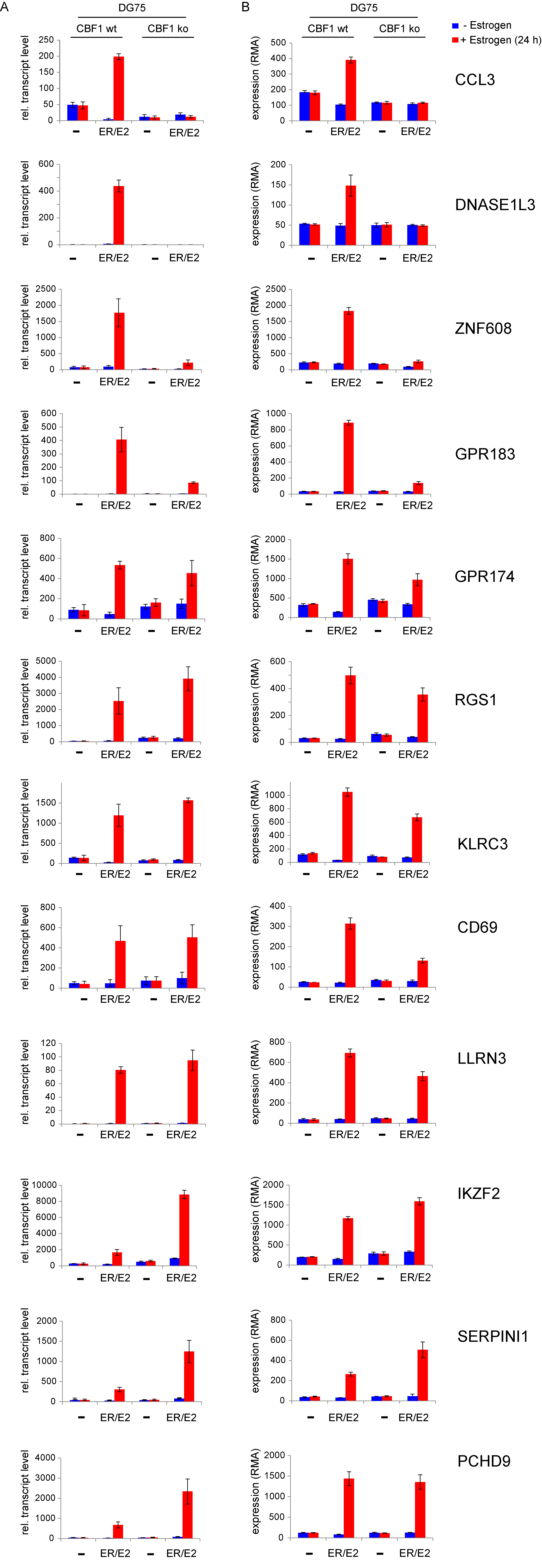

Supplement: S4 Fig — (A) Relative transcript levels of EBNA2 target genes were quantified from total RNA samples of the indicated cell lines by RT-qPCR. All results were normalized to actin B transcript levels. (B) For comparison the expression levels measured by gene array hybridization are shown in parallel. (TIF) [file ppat.1006664.s004.tif]

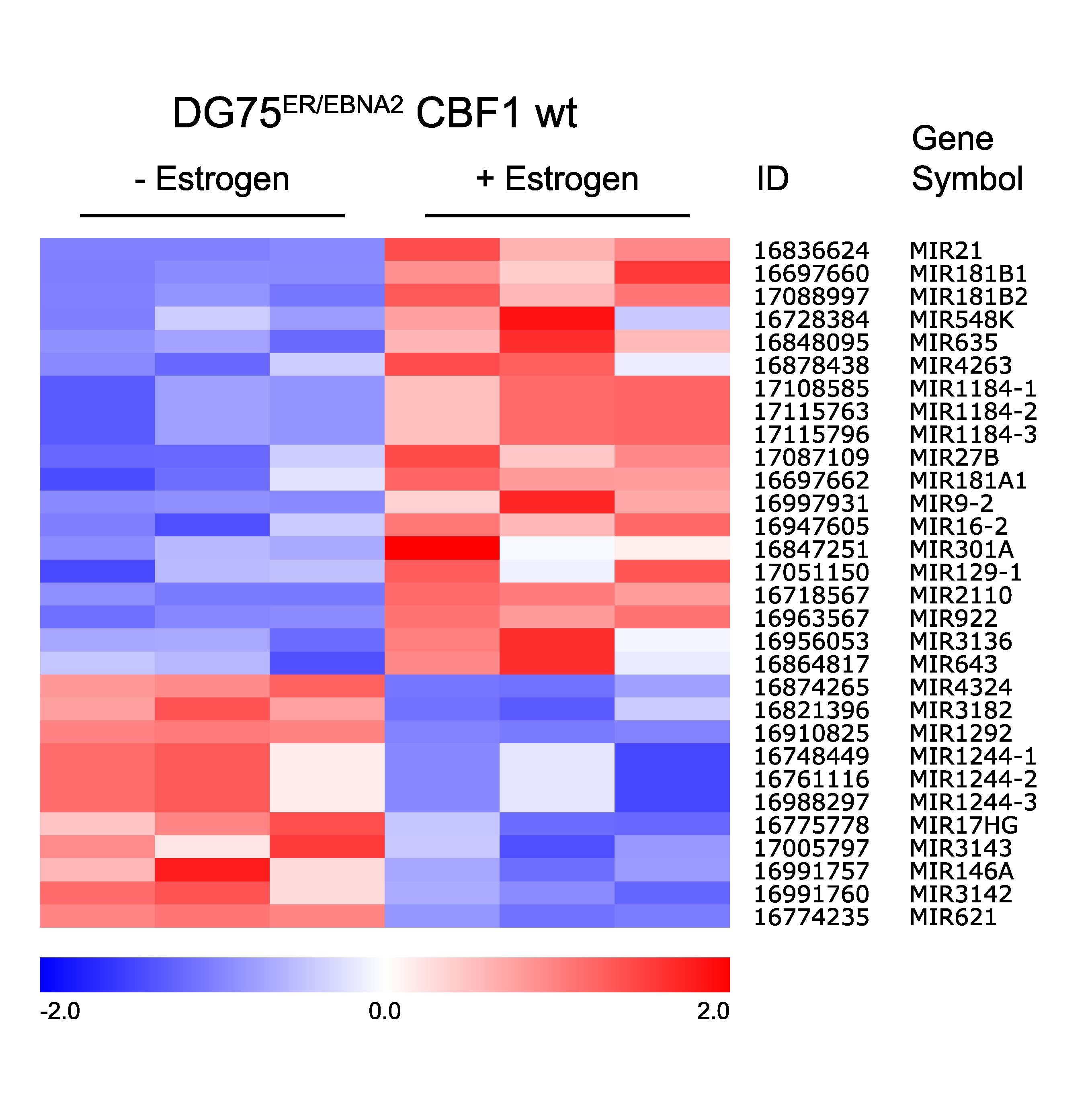

Supplement: S5 Fig — (TIF) [file ppat.1006664.s005.tif]

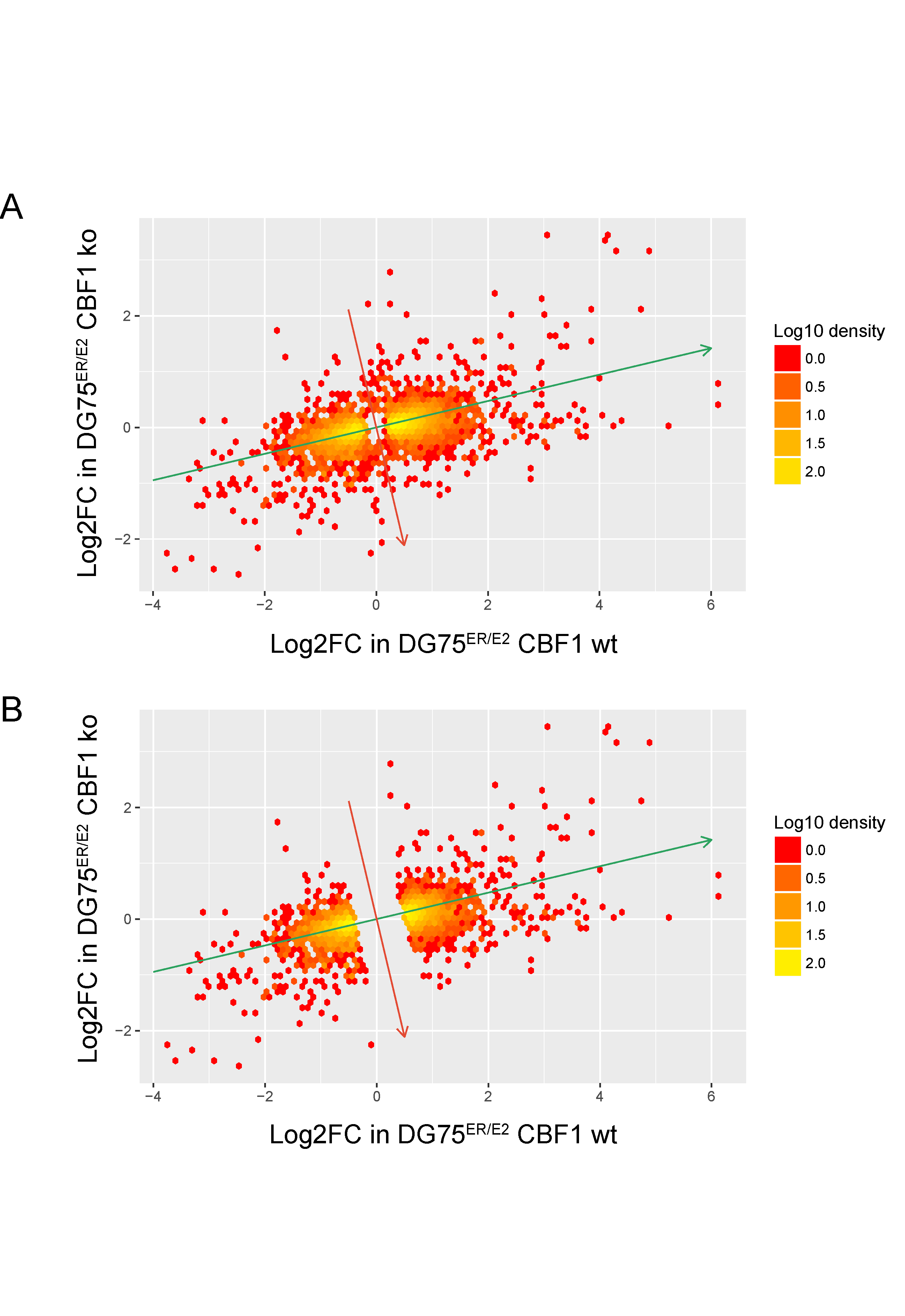

Supplement: S6 Fig — Since on average target gene expression changes in CBF1 positive cells were stronger than in CBF1 negative cells, principle component analysis on EBNA2 regulated genes was used to identify specific subpopulations: The first principle component (green arrow) describes the upregulation of genes in both cell lines, the second principle component (red arrow) describes the degree of CBF1 dependence. The scatter blots depict all genes (A) or the top 2000 (B) induced/repressed genes which are regulated in at least one cell line. (TIF) [file ppat.1006664.s006.tif]

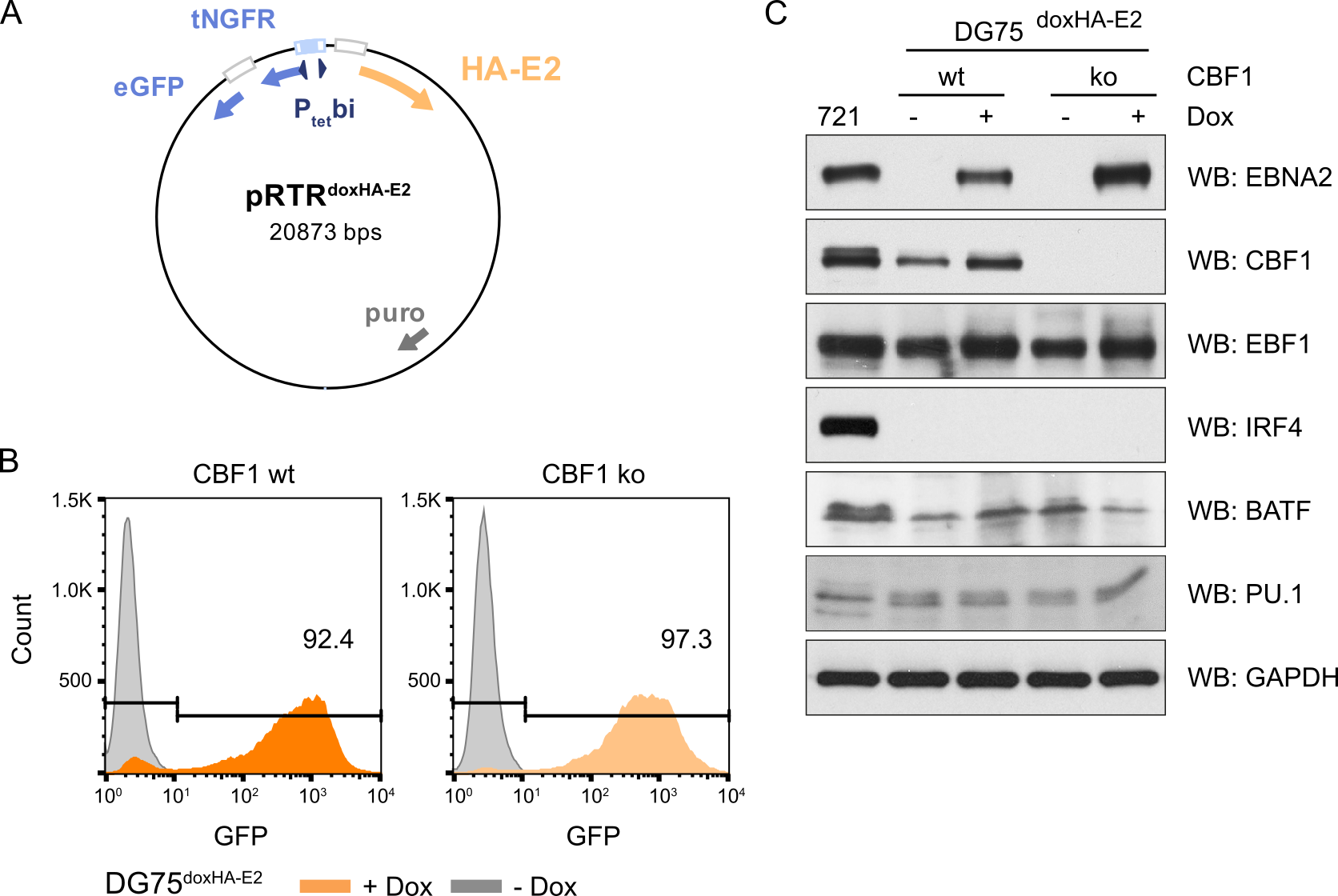

Supplement: S7 Fig — (A) pRTRdoxHA-E2 vector used to generate stable DG75 cell lines. The coding sequence for EBNA2 fused to a N-terminal HA-tag (HA-E2), plus a preceding intron of the beta-globin gene for enhanced expression, was cloned into the pRTR vector [69, 70] using SfiI restriction sites. The bidirectional promoter simultaneously drives the expression of HA-EBNA2 and the bicistronic reporter construct consisting of a truncated nerve growth factor receptor gene (tNGFR) and enhanced green fluorescent protein (eGFP) gene upon doxycycline induction. (B) Expression of HA-EBNA2 was induced with 1 μg/ml doxycycline (Dox) for 24 h and monitored by quantifying eGFP expression via flow cytometry and scored at least 89% with a maximum of 5% difference between DG75 CBF1 wt and ko cells. Data from one representative experiment (n = 3) and percentages of induced cells are shown. (C) Western Blot analysis confirming the expression of HA-EBNA2 in DG75doxHA-E2 cell lines 24 h post induction with 1 μg/ml Dox. 721 is an LCL cell line used as positive control for all western blots. The absence of CBF1 expression in the DG75doxHA-E2 CBF1 ko cell line is confirmed. EBF1, IRF4, BATF, and PU.1/SPI1 are shown for comparison. GAPDH serves as loading control. For all blots 30μg protein lysate was used for all lanes with a single exception: For EBNA2 blots 3 μg DG75 lysates and 30μg 721 lysate were used in order to take the 5–10 fold higher EBNA2 expression of DG75 into consideration. (TIF) [file ppat.1006664.s007.tif]

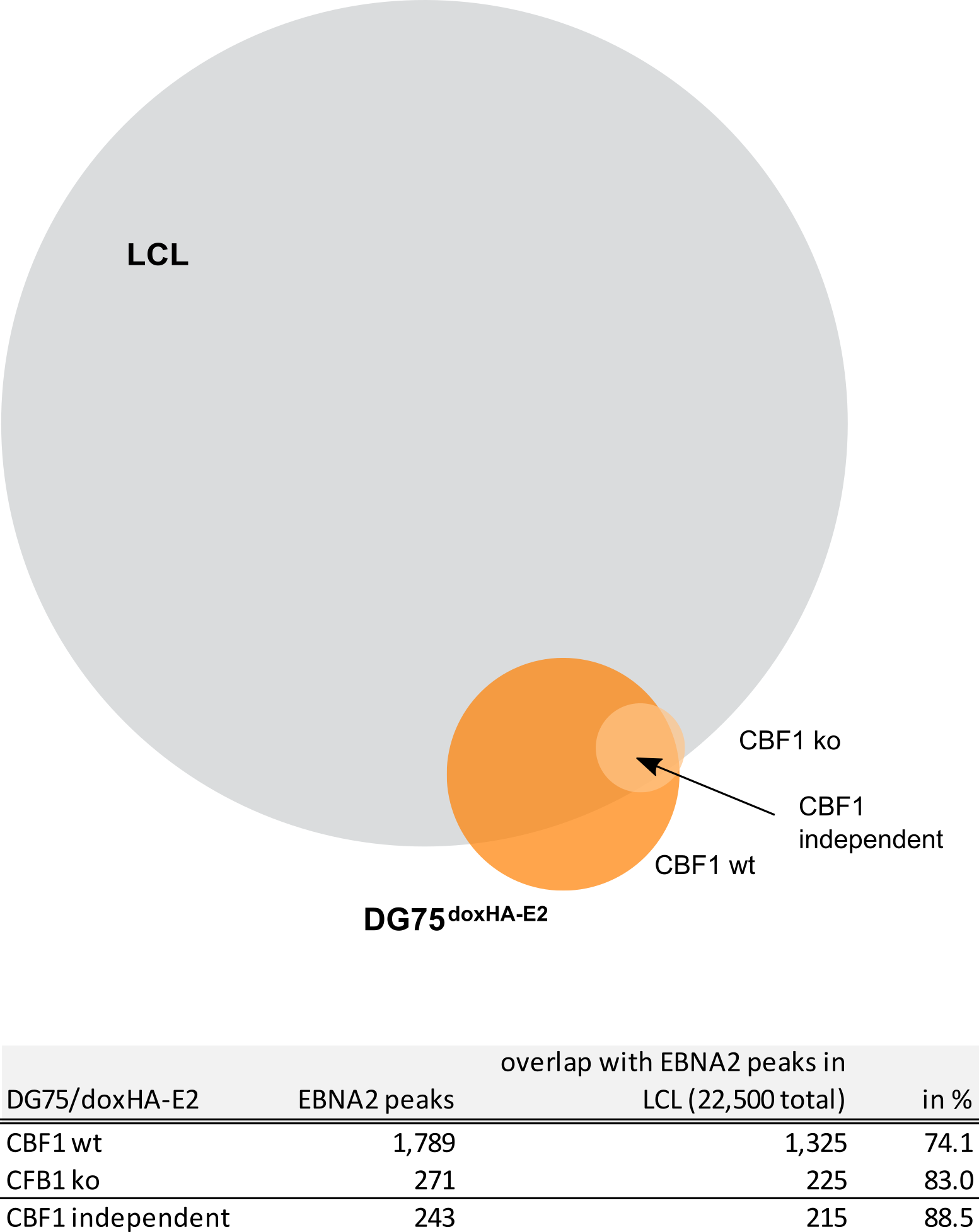

Supplement: S8 Fig — The table lists (lower part) the called peaks and the fraction of peaks which also score in LCL EBNA2. (TIF) [file ppat.1006664.s008.tif]

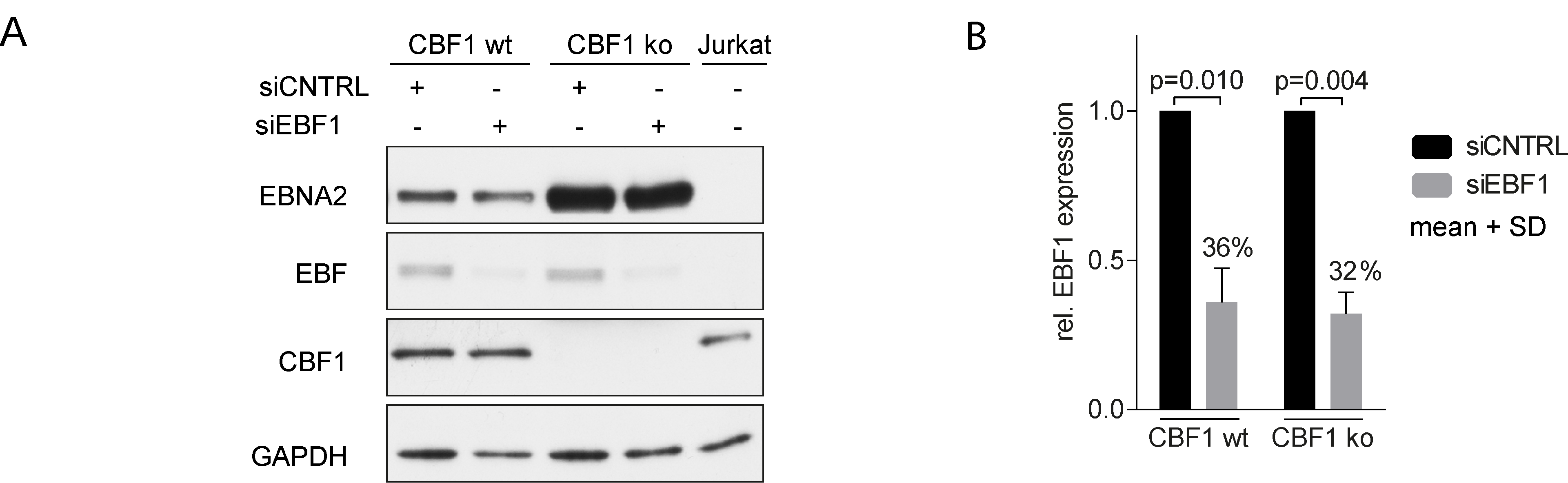

Supplement: S9 Fig — DG75doxHA-E2 CBF1 wt or CBF1 ko B cells were transfected with a mixture of scrambled non-targeting siRNAs (siCNTRL) or EBF1 specific siRNAs (siEBF1). 8 h post transfection, EBNA2 transcription was induced. 24 h post transfection, cells were harvested and analyzed by immunoblots and ChIP-qPCR. (A) Representative immunoblots showing expression levels of EBNA2, EBF1, CBF1, and GAPDH before and after knockdown (n = 3). EBF1 negative Jurkat cell lysate served as a negative control. (B) Protein band intensities were quantified by densitometry. The change of EBF1 protein expression in siRNA (siEBF1) treated compared to non-treated cells (CNTRL) is significant according to paired t-test when indicated. (TIF) [file ppat.1006664.s009.tif]
