# Supplementary material for: EBF1 binds to EBNA2 and promotes the assembly of EBNA2 chromatin complexes in B cells
Source: PLoS Pathog. 2017 Oct 2;13(10):e1006664. doi: 10.1371/journal.ppat.1006664 (PMC5638620; doi:10.1371/journal.ppat.1006664)
Supplement: S3 Table — (PDF) [file ppat.1006664.s012.pdf]

S3 Table qPCR Primer

(A) RT-qPCR Primer

Gene	Sequence
CCL3	Forward primer: ATGCAGGTCTCCACTGCTG<br>Reverse primer: TTTCTGGACCCACTCCTCAC
DNase1L3	Forward primer: AGGACACCACGGTGAAGAAG<br>Reverse primer: GTGAAGGCCCTTGAAGACTG
ZNF608	Forward primer: TTCAGGGCTCATGGGAAACT<br>Reverse primer: GACACTCTGGCTCTGTCACT
GPR183	Forward primer: ACTACACAGAGACCCGAACG<br>Reverse primer: CCACGAGCCCAATGATGAAG
GPR174	Forward primer: TGTACCCCTTTCGCTTCCAT<br>Reverse primer: ACAAACCCAATCAACTCGCC
RGS1	Forward primer: TGCAATGGTCTCAATCTCTGG<br>Reverse primer: CAGGGCAAAAGATCAGACTCTG
KLRC3	Forward primer: TCTGGCCAGCATTTTACCTTC<br>Reverse primer: CCACGTACATGTAGCATTGCA
CD69	Forward primer: AACACGTCATGAAGGGTCCT<br>Reverse primer: GGCTGTCTGATGGCATTGAG
LLRN3	Forward primer: GAGGTGAAGCATTCCACAGA<br>Reverse primer: CCCATGCTTCTTCAGTATTTGC
IKZF2	Forward primer: TGAAATGTGACGTCTGTGGC<br>Reverse primer: TTGAACGGCTTCTCTCCAGA
SERPINI1	Forward primer: AGACGAAAGCAGGAACGAGA<br>Reverse primer: GCCCCAAGTTCCATCATTCC
PCDH9	Forward primer: TGGGAAAGGGGAAAGGTTGA<br>Reverse primer: TCAGAGCAGCCAACAGGTAA

(B) ChIP-qPCR Primer

	Locus	Sequence
**CBF1-independent loci**	chr19:42,633,923-42,634,005	Forward primer: TGGCCTCAAGGGAGTGAAC<br>Reverse primer: GGCCACCTCTCTTGTTGTG
	chr16:11,043,370-11,043,449	Forward primer: AGTGCACATTGGCAGAACTC<br>Reverse primer: CTCTGAGCTTGGGTCCTCAC
	chr3:39,189,615-39,189,702	Forward primer: TAGGCAAGCGTGAGAGAAGC<br>Reverse primer: CCCGGAAACATCTGTGAGTC
**CBF1-dependent loci**	chr4:40,187,701-40,187,810	Forward primer: AGGGAAAGTTAAGACAGGCCTT<br>Reverse primer: TAGAGGATTCCAACCCAATGCC
	chr10:74,057,129-74,057,245	Forward primer: CTTGGCACGAACTCTTCCTTC<br>Reverse primer: AGATGTGAGAATGATGTGGCCG
	chr11:9,623,169-9,623,267	Forward primer: GCTGGGTTTGCGGTTTAATG<br>Reverse primer: TTGCTGCTCCCTAAGGTTTG
**Control loci**	(+) chr8:24224967-24225059	Forward primer: CTTCATGGCTACAGACTCTTGG<br>Reverse primer: CCTATGTCTCGCTTCCTGCT
	(-) chr8:99055763-99055882	Forward primer: CTGGTCTGACGCTCCTGACT<br>Reverse primer: CAGTGCCCAGAATTCCAGAT
