# Supplementary material for: EBF1 binds to EBNA2 and promotes the assembly of EBNA2 chromatin complexes in B cells
Source: PLoS Pathog. 2017 Oct 2;13(10):e1006664. doi: 10.1371/journal.ppat.1006664 (PMC5638620; doi:10.1371/journal.ppat.1006664)
Supplement: S4 Table — (PDF) [file ppat.1006664.s013.pdf]

S4 Table Summary ChIP-Seq Results

(A) E2 ChIP-seq in DG75 cell lines - Reads after different workflow steps and mapping to the human genome

DG75 Cell Line	Replicate - Sample Type	Read Count			Internal Designation
		Demultiplexed	Mappable (% of Demultiplexed)	Uniquely Mappable (% of Demultiplexed)	
CBF1 wt	E2-I-ChIP	17,455,101	94.81	69.06	LG620_wt_E2
	E2-I-input	19,901,128	97.69	70.21	LG620_wt_input
	E2-II-ChIP	34,613,332	94.78	68.89	LG625_wt_E2
	E2-II-input	27,927,224	97.93	70.56	LG625_wt_input
CBF1 ko	E2-I-ChIP	17,320,583	97.15	69.82	LG620_ko_E2
	E2-I-input	20,294,961	97.48	69.64	LG620_ko_input
	E2-II-ChIP	25,601,620	97.21	70.81	LG625_ko_E2
	E2-II-input	29,324,523	97.66	70.28	LG625_ko_input

Reads obtained after demultiplexing were directly subjected to Bowtie2 software for mapping to the human genome (hg19).

(B) E2 ChIP-seq in DG75 cell lines - Peaks identified using MACS2

DG75 Cell LIne	Subjected to MACS2	Read Count		Allowed Duplicate Tags	Redundancy Rate (%)	E2 Peaks
		Merged Mapped Reads	Filtered			
CBF1 wt	E2-ChIP	49,354,861	48,526,538	2	1.68	**1,937**
	E2-input	46,790,793	45,788,697	2	2.14	
CBF1 ko	E2-ChIP	41,714,810	41,006,646	2	1.70	**429**
	E2-input	48,423,478	47,262,377	2	2.40	

Mapped reads of replicates were merged and subjected to MACS2 peak calling algorithm. Here reads were filtered for allowed duplicate tags, which represent maximum permitted reads mapping to the exact same position. This value is calculated by MACS2 in accordance with absolute read count and genome coverage. The redundancy rate is indicating the percentage of duplicate reads not allowed and displays a measurement for library complexity.

(C) E2 ChIP-seq in DG75 cell lines - Signal and mappability corrected peaks

DG75 Cell Line	Identified by MACS2	Signal corrected	Blacklist corrected	GM12878 compatible	% of MACS2 peaks
**CBF1 wt**	1,937	1,818	1,793	**1,789**	92.4
**CBF1 ko**	429	286	271	**271**	63.2

Peaks identified by MACS2 were further filtered to exclude peaks which display a negative amplitude, fall on blacklisted regions or a chromosome not compatible with GM12878, the LCL used by ENCODE.

(D) Reads after different workflow steps and mapping

ChIP	Replicate - Sample Type	Read count			
		Demultiplex	Trimming (% of Demultiplexed)	Mappable Reads (% of Trimmed)	Uniquely Mappable Reads (% of Trimmed)
**E2**	E2-I-ChIP	21,451,466	21,321,357 (99.4)	95.8	71.3
	E2-I-input*	35,575,701	35,502,629 (99.8)	98.8	70.1
	E2-II-ChIP	26,478,900	26,212,962 (99.0)	96.5	70.0
	E2-II-input	29,618,835	29,558,726 (99.8)	98.8	72.0

Reads obtained after demultiplexing were subjected to trimming (percentages of remaining reads are indicated) and subsequently to Bowtie2 for mapping to the human genome (hg19). * E2-I-input and E3C-II-input are actually the same sample since E2 and Flag-E3C ChIPs were performed using the same chromatin preparation.

(E) Peaks identified in the human genome using MACS2

ChIP	Subjected to MACS2	Read Count		Allowed Duplicate Tags	Redundancy Rate (%)	Peaks
		Merged Mapped Reads	Filtered			
**E2**	E2-ChIP	45,731,868	44,783,737	2	2.1	**23,314**
	E2-input	64,285,263	63,034,015	2	1.9	

Mapped reads of replicates were merged and subjected to MACS2 peak calling algorithm. Here reads were filtered for allowed duplicate tags, which represent maximum permitted reads mapping to the exact same position. This value is calculated by MACS2 in accordance with absolute read count and genome coverage. The redundancy rate is indicating the percentage of duplicate reads not allowed and displays a measurement for library complexity.

(F) Signal and mappability corrected peaks in the human genome

ChIP	Identified by MACS2	Signal corrected	Blacklist corrected	GM12878 compatible	% of MACS2 peaks
**E2**	23,314	22,857	22,715	**22,500**	96.5

Peaks identified by MACS2 were further filtered to exclude peaks which display a negative amplitude, fall on blacklisted regions or a chromosome not compatible with GM12878, the LCL used by ENCODE.

(G) E2 binding sites identified in LCL and DG75 cells using MACS2

E2 peaks	LCL	DG75
total	22,500	1,789
**shared peaks for each subset****	1,227	1,325*
unique	21,274	464

* EBNA2 peak subset "LCL/DG75doxHA-E2 shared" which was used for further analyses (Fig. 2).
**The numerical discrepancy between the two shared subsets is caused by the fact that neighboring peaks in LCLs might score as a single peak in DG75 and vice versa.
