# Supplementary material for: EBF1 binds to EBNA2 and promotes the assembly of EBNA2 chromatin complexes in B cells
Source: PLoS Pathog. 2017 Oct 2;13(10):e1006664. doi: 10.1371/journal.ppat.1006664 (PMC5638620; doi:10.1371/journal.ppat.1006664)
Supplement: S5 Table — (PDF) [file ppat.1006664.s014.pdf]

S5 Table Public Resources Used for this Study

(A) TF and histone modification ChIP-seq experiments by the ENCODE project used in this study

ChIP	Experiment Name	Narrow Peaks (bed)	Broad Peaks (bed)	ENCODE Reanalyzed	Lab	Release Date	Antibody
SPI1/PU.1	ENCSR000BGQ	-	ENCFF001TYN, ENCFF001TYO, ENCFF001TYP	**ENCFF002CHQ**	R. Myers, HAIB	18.07.11	ENCAB000AKF
EBF1	ENCSR000DZQ	ENCFF001VEF	-	**ENCFF002COS**	M. Snyder, Stanford	29.10.11	ENCAB000AFX
BATF	ENCSR000BGT		ENCFF001TWG ENCFF001TWH	**ENCFF002CGQ**	R. Myers, HAIB	18.07.11	ENCAB000AED
IRF4	ENCSR000BGY		ENCFF001TXH	**ENCFF002CHB**	R. Myers, HAIB	18.07.11	ENCAB000AHZ
H3K27ac	ENCSR000AKC	-	ENCFF001SUG	-	B. Bernstein, Broad	10.02.11	ENCAB000ANA
H3K4me1	ENCSR000AKF	-	ENCFF001SUE	-	B. Bernstein, Broad	10.02.11	ENCAB000ADW
H3K4me3	ENCSR000AKA	-	ENCFF001SUF	-	B. Bernstein, Broad	10.02.11	ENCAB000BLJ

ChIP-seq data for TFs and histone modifications used in this study are listed. The information including experimental procedures and all submitted files can be found at www.encodeproject.org. Peak files (bed) which were used for e.g. cluster analyses are highlighted in bold letters. Signal tracks published by the ENCODE project were not used in this thesis, since they are not normalized to input samples. To correct for input reads, signal tracks from aligned reads (bam files) were generated.

(B) Accession numbers for data published by other laboratories used in this studies

Name	Cells Line	Data Deposit Platform	Accession No. (Experiment)	Accession No. (Sample)	ENCODE experiment	Description	Publication
**H3K4me1**				GSM1027296	-	ChIP-seq	
**H3K4me3**	**CD19+ primary cells**	GEO	GSE18927	GSM1027300	-	ChIP-seq	Bernstein et al. (2010)
**H3K27ac**				GSM1027287	-	ChIP-seq	
**Input**				GSM1027304	-	ChIP-seq control	
**H3K4me1**				ERX297414	-	ChIP-seq	
**H3K4me3**	**DG75**	EMBL-EBI European Nucleotide Archive (ENA)	PRJEB1912 (study) ERS333899 (sample = DG75)	ERX297407	-	ChIP-seq	Kretzmer et al. (2015)
**H3K27ac**				ERX297417	-	ChIP-seq	
**Input**				ERX297450	-	ChIP-seq control	

ChIP-seq data used in this studies and sources are listed. For all listed experiments fastqsanger files were downloaded, reads were mapped and re-analyzed.
